# Anti-inflammatory 25(OH)D_3_, a natural steroid hormone, may complement all-trans retinoic acid therapy for differentiation syndrome in acute promyelocytic leukemia

**DOI:** 10.1038/s41419-025-08109-7

**Published:** 2025-11-03

**Authors:** Károly Jambrovics, Wedean Al-Hadban, Anett Mázló-Türk, Istvan Szatmari, Boglárka Dobó, Gyula Reményi, Ádám Jóna, Gábor Koncz, Zoltán Balajthy

**Affiliations:** 1 Department of Biochemistry and Molecular Biology, Faculty of Medicine, University of Debrecen, Debrecen, Hungary; 2 Doctoral School of Molecular Cell and Immune Biology, Faculty of Medicine, University of Debrecen, Debrecen, Hungary; 3 Department of Immunology, Faculty of Medicine, University of Debrecen, Debrecen, Hungary; 4 Department of Hematology, Faculty of Medicine, University of Debrecen, Debrecen, Hungary; 5 Doctoral School of Clinical Medicine, University of Debrecen, Debrecen, Hungary

**Keywords:** Acute myeloid leukaemia, Leukaemia

## Abstract

Differentiation syndrome (DS) is a serious complication with an unclear pathogenesis that arises following all-trans retinoic acid (ATRA) or arsenic trioxide induction therapy in acute promyelocytic leukemia (APL). DS symptoms include pyrexia, respiratory compromise, increased body mass, fluid accumulation, pulmonary infiltrates, hypotension, tachycardia, edema, and sepsis. It can also affect the kidneys and the central nervous system. The standard treatment to counteract DS involves the temporary cessation of ATRA or arsenic trioxide treatment and the administration of high-dose steroids to mitigate the inflammatory response. If left untreated, DS can be fatal. Further research has revealed that the inability to promptly recognize intracranial hemorrhage (ICH) in patients with APL may result in lethal consequences, with the cytokine storm identified as the principal factor in this scenario as well. ATRA therapy is known to induce transglutaminase 2 (TG2), which functions as a catalyst for DS development. In this study, we used NB4 cell lines and cells from a human patient with APL to investigate the ex vivo effects of ATRA and 25(OH)D_3_ cotreatment on NF-κB-regulated luciferase reporter gene activity. ATRA alone substantially enhanced cellular NF-κB luciferase reporter gene activity, whereas 25(OH)D_3_ dose-dependently reduced this activity. During ATRA-initiated cell maturation, 25(OH)D_3_, known as calcidiol, suppressed the mRNA expression of NF-κB (*p65/p50*) and Rel family members, as well as the expression of genes associated with increased NF-κB activity. 25(OH)D_3_ also inhibited the ATRA-induced production of cytokines (e.g., IL-8), including IL-1β, TNF-α, and MCP-1, associated with the “cytokine storm.” Combined treatment with ATRA plus 25(OH)D_3_ reduced cellular phospho-p65 and transglutaminase 2 (TG2) levels and increased the level of inhibitor of Rel (IκB), thereby attenuating the cytokine storm. These findings provide a molecular interpretation for clinical DS and IHC observations and may support future exploration of ATRA plus 25(OH)D_3_ cotreatment as a therapy for APL.

## Introduction

Acute promyelocytic leukemia (APL), a type of cancer characterized by granulopoiesis differentiation arrest, was the first human cancer to be successfully treated with all-trans retinoic acid (ATRA), a cell differentiation inducer [1]. In the NB4 WT acute promyelocytic leukemia cell line, ATRA treatment induces the maturation of cells into neutrophil granulocytes, resulting in the expression of multiple genes, including transglutaminase 2 (TG2), which is not typically expressed in healthy neutrophil granulocytes [2–4]. However, although ATRA therapy induces this cell differentiation in patients with APL, approximately 30% of patients experience differentiation syndrome (DS), which is a severe complication in individuals receiving targeted therapies (e.g., ATRA or arsenic trioxide) and a cause of death in 1–5% of APL patients [5, 6].

DS is common in the early stages of treatment but can also manifest during subsequent phases. It is triggered by the differentiation of leukemic cells into cell types that produce inflammatory mediators and cytokines that can affect multiple organ systems. In addition, a recent study indicated that failure to identify intracranial hemorrhage (ICH) promptly in patients with APL may result in fatal outcomes. In this case, the cytokine storm was deemed the primary cause of induction therapy failure and early mortality [7].

One agent that has shown benefits in the treatment of various cancer types is vitamin D (1,25-(OH)2D_3_; calcitriol) [8]. The anti-inflammatory properties of 1,25(OH)_2_D_3_ are attributed to the expression and stabilization of IκBα (NF-κB inhibitory protein α), which suppresses NF-κB activity and reduces NF-κB protein levels in various cell types. These inhibitory effects are significant for medical oncology, as NF-κB is essential in cancer pathogenesis and is constitutively expressed in malignant tumors. An interaction between the vitamin D receptor and IκB kinase β has been shown to prevent NF-κB binding to DNA [9, 10]; however, further research is needed to establish a conclusive association [11–13].

Another factor that has recently emerged as essential for the survival of a various of cancer cell types is TG2, an enzyme that plays a crucial role in various physiological processes in the human body, including differentiation, apoptosis, phagocytosis, signal transduction, adhesion, wound healing, and angiogenesis [14, 15]. It also facilitates posttranslational modifications, such as protein crosslinking, amine integration, acylation, deamidation, and self-cleavage of its own output. TG2 is found in various cell types and compartments and acts as a scaffold to bind growth factor receptors, integrins, fibronectin, and syndecan-4 to the extracellular matrix. The discovery that TG2 is a GTP/GDP-binding protein that hydrolyzes GTP and functions as a G protein has also led to its designation as a key factor in cancer cell survival. Currently, evidence supports a role for TG2 in the activation of signaling pathways that drive drug resistance, cancer stem cell survival, metastasis, inflammation, epithelial-mesenchymal transition (EMT), and angiogenesis [16, 17].

We have previously shown that NF-κB-dependent and TG2-dependent expression of cytokines and chemokines, such as TNF-α, IL-1β, MCP-1/CCL2, and MDC/CCL22, occur during the ATRA-induced differentiation of NB4 WT cells [4, 18, 19]. In the present study, we demonstrated that ATRA alone can significantly increase the activity of an NF-κB luciferase reporter gene in cells obtained from a human patient with APL. We also showed that the APL cells respond similarly to NB4 WT cells when subjected to ATRA and that elevated TG2 levels are accompanied by the onset of a cytokine storm. Of particular interest, the addition of 25(OH)D_3_ reduced the leukemic cytokine burden by diminishing NF-κB phospho-p65 and TG2 levels while increasing inhibitor of Rel (IκB) levels. Our results suggest that a combined therapy that includes both ATRA and 25(OH)D_3_ could be beneficial in the treatment of APL and the prevention of DS.

## Materials and methods

### Isolation and culture of healthy human neutrophil granulocytes and APL cells from patients’ peripheral blood

Heparinized leukocyte-enriched buffy coats were obtained from healthy blood donors at the Regional Blood Center of the Hungarian National Blood Transfusion Service, Hungary, with written approval from the director and the Regional and Institutional Research Ethical Committee of the University of Debrecen, Faculty of Medicine. APL cells from human peripheral blood were obtained from a patient with APL diagnosed by the In Vitro Diagnostic Center of the University of Debrecen. The APL cells were isolated via Ficoll-Paque (Merck) density gradient centrifugation and cryopreserved in fetal bovine serum (Gibco, Paisley, Scotland) containing 10% dimethyl sulfoxide (DMSO; Sigma‒Aldrich-Merck) by storage in liquid nitrogen. Patient permission was acquired in accordance with the Declaration of Helsinki (DE RKEB/IKEB 5823-2021).

### Statistical analysis

Statistical analyses were carried out using GraphPad Prism version 9.1.8 with a two-way ANOVA multiple comparison test (Bonferroni post hoc test; *p* < 0.05, *p < 0.01, and **p < 0.001).

### Additional methods

All experiments were conducted using worldwide accepted laboratory protocols. The methods for

(i) Isolation and culture of human neutrophils from peripheral blood,
(ii) Transduction of NB4 and APL patient cells with a luciferase response element and activity measurement,
(iii) Flow cytometry,
(iv) ELISA and ELISA PLEX,
(v) TaqMan™ Array Human NF-κB Pathway,
(vi) Immunoblotting and RT-qPCR, are detailed in the Supplementary Section.

### Ethics approval

All the methods were performed in accordance with the relevant guidelines and regulations. The approval for the experiments has been obtained from the Regional and Institutional Research Ethical Committee of the University of Debrecen, Faculty of Medicine. Patient permission was acquired in accordance with the Declaration of Helsinki (DE RKEB/IKEB 5823-2021). Hereby, we state that informed consent was obtained from all the participants.

## Results

### 25(OH)D_3_ administration reduces NF-κB transcriptional activity without significantly affecting the survival or number of ATRA-differentiated NB4 cells

**Fig. 1:**
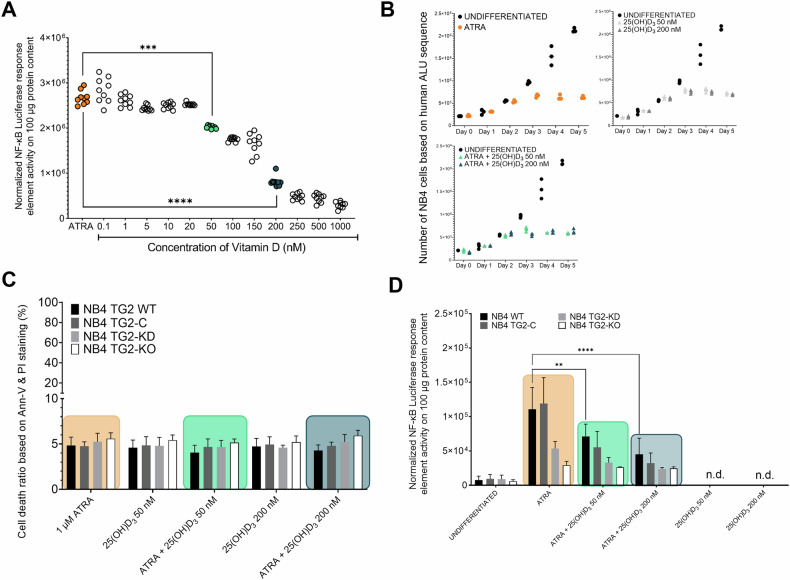
25(OH)D_3_ administration reduces NF-κB pathway activity without altering cell survival or cell numbers. **A** 25(OH)D_3_ administration reduces NF-κB pathway activity without altering cell survival or cell numbers. A NF-κB response element-driven luciferase activity in NB4 WT cells treated with 1 µM ATRA or 1 µM ATRA plus increasing nanomolar (nM) concentrations of 25(OH)D_3_ for five days. The relative light units (RLU) of luciferase reporter activity in treated and harvested cells were measured in triplicate. The graph represents the mean RLU values ± S.D. (*n* = 5). **B** Total APL cell numbers were quantified using a human ALU-based RT‒qPCR method. Cells were treated with 1 µM ATRA, 1 µM ATRA + 50 nM 25(OH)D_3_ or 1 µM ATRA + 200 nM 25(OH)D_3_. Cell counts were measured in triplicate over five days (*n* = 5). **C** Cell viability of NB4 cell lines treated with 1 µM ATRA, 1 µM ATRA + 50 nM 25(OH)D_3_, or 1 µM ATRA + 200 nM 25(OH)D_3_. The percentage of viable cells was determined based on Annexin V and propidium iodide (P.I.) staining using flow cytometry (Flowing Software 1.02). The graph shows mean values ± S.D., measured in triplicate on day 5 (*n* = 5). **D** NF-κB response element-driven luciferase activity in NB4 cell lines treated with 1 µM ATRA or 1 µM ATRA plus increasing nM concentrations of 25(OH)D_3_ for five days. Luciferase activity (RLU) was measured in treated and harvested cells. The graph represents mean RLU values ± S.D. on day 5 (*n* = 5). The color codes: ATRA (orange), cotreatment with ATRA and lower concentrations of 25(OH)D3 (green), and cotreatment with ATRA and higher concentrations of 25(OH)D3 (dark green). Statistical significance was determined using two-way analysis of variance (ANOVA) with Bonferroni post hoc test (*p < 0.05, **p < 0.01, ***p < 0.001, ****p < 0.0001). Colored frames indicate different treatments throughout the experiment.

Treatment of NB4 WT cells with ATRA significantly increased NF-κB luciferase reporter gene activity. Cotreatment with 25(OH)D_3_ reduced this luciferase activity in a dose-dependent manner, decreasing it by nearly 90% at 1 µM (400 ng/mL) and by approximately two-thirds at 200 nM (Fig. 1A). Annexin-V and propidium iodide double labeling of cells treated with ATRA and 25(OH)D_3_ (50 or 200 nM) demonstrated no significant differences in cell numbers compared to ATRA treatment alone (Fig. 1C). To investigate the presence of TG2 on the cell survival of the NB4 cells, we also introduced the 25(OH)D_3_ vitamin treatment to cell lines in which the level of the TG2 is attenuated (TG2 knockdown) and fully eliminated (TG2 knockout cell line generated by TALEN technology). In the further experiments we used these four distinct TG2-expressing cell lines (NB4 WT, TG2-C [virus control], TG2-KD [TG2 knockdown], and TG2-KO [TG2 knockout]) confirmed that adding 25(OH)D_3_ (50 or 200 nM) did not significantly affect cell viability, indicating that cell death was independent of TG2 levels (Fig. 1C). Assessment of NF-κB response element-driven luciferase reporter gene activity in the four cell types following ATRA and 25(OH)D_3_ treatment revealed an approximately 35% reduction in NF-κB reporter gene activity when 50 nM 25(OH)D_3_ was added, compared to ATRA treatment alone. This reduction increased to approximately 65% with 200 nM 25(OH)D_3_ (Fig. 1D). These decreases were largely TG2 dependent, as NB4 TG2-KO cells showed no significant reduction in NF-κB response element activity in response to 25(OH)D_3_. Generally, ATRA-treated NB4 TG2-KO cells exhibited 75% lower reporter gene activity than ATRA-treated NB4 WT cells, regardless of the 25(OH)D_3_ concentration within the tested range (Fig. 1D). Overall, these results demonstrate that the ATRA-induced increase in NF-κB transcriptional activity is TG2 dependent and can be effectively suppressed by the administration of 25(OH)D_3_.

### mRNA expression of NF-κB and Rel family genes associated with increased NF-κB activity is inhibited by 25(OH)D_3_

**Fig. 2:**
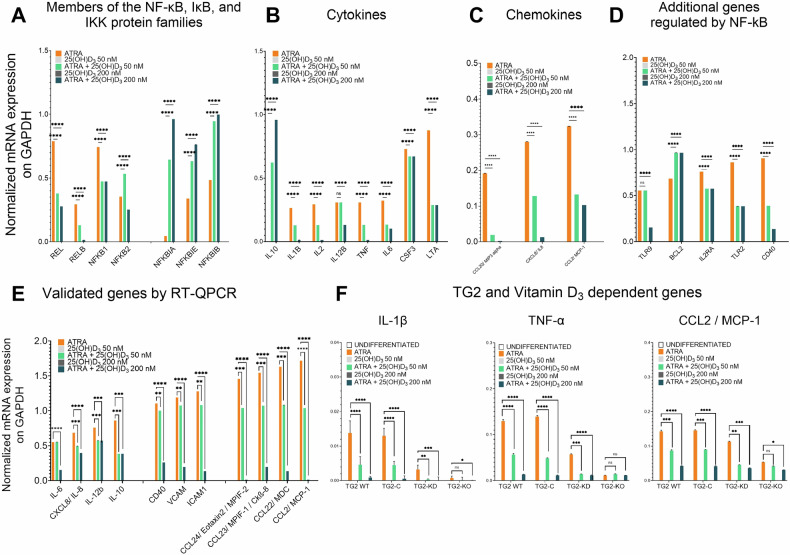
Gene expression of the NF-κB family is associated with increased NF-κB activity, which is inhibited by 25(OH)D_3_. **A–D** NB4 WT cells were treated with 1 µM ATRA, 50 nM 25(OH)D_3_, 1 µM ATRA + 50 nM 25(OH)D_3_, 200 nM 25(OH)D_3_, or 1 µM ATRA + 200 nM 25(OH)D_3_ for 5 days. On day 5, cells were harvested and total RNA was isolated using the TRIzol method. cDNA generated via RT-PCR was subsequently used for RT-qPCR targeting NF-κB pathway member genes (TaqMan™ Array Human NF-κB Pathway). **E** Quantitative gene expression analysis was performed for selected NF-κB target genes to validate the array results. The graphs show the relative mRNA expression values in NB4 WT cells. **F** mRNA expression levels of IL-1β, TNF-α, and CCL2/MCP-1 in NB4 cell lines (NB4 WT, TG2-C, TG2-KD, and TG2-KO) following cotreatment with ATRA and 25(OH)D3. Expression values were calculated using the ^ΔΔ^Ct method. Statistical significance was determined via two-way analysis of variance (two-way ANOVA; Bonferroni post hoc test; *p < 0.05, **p < 0.01, ***p < 0.001, ****p < 0.0001) (*n* = 5).

We used the TaqMan^TM^ Array Human NF-κB Pathway to further explore the effects of cotreatment of NB4 WT cells with ATRA and 25(OH)D_3_ on NF-κB-regulated genes involved in inflammation. Four gene classes showed altered gene expression in response to 25(OH)D_3_. The first class included transcription factors and NF-κB transcription inhibitors, and the cotreated cells showed decreased expression of *REL, RELB, NFkB1*, and *NFkB2*, and increased expression of transcriptional inhibitory genes (*NFkBIA, NFkBIE*, and *NFkBIB*) (Fig. 2A). The second class included *IL-10* and the ATRA-upregulated genes *IL-1B, IL-2, IL-12B, TNF-α, IL-6, CSF3*, and *LTA*. Cotreated cells showed significantly reduced expression of all the ATRA-upregulated genes, except for *CSF2* (Fig. 2B). The third group included the ATRA-upregulated chemokine genes *CCL20/MIP3-alpha, CXCL8/IL-8*, and *CCL2/MCP1*. Cotreated cells exhibited significantly reduced expression of these genes (Fig. 2C). The fourth group included genes containing NF-κB response elements and noninflammatory genes, such as *TLR9, BCL2, IL-2RA, TLR2*, and *CD40*. The cotreated cells showed reduced expression of all these genes, except for *BCL2* (Fig. 2D).

Real-time quantitative polymerase chain reaction (RT‒qPCR) analysis of the expression patterns of 11 target genes regulated by NF-κB confirmed that 25(OH)D_3_ significantly attenuated gene expression in ATRA-treated NB4 cells (Fig. 2E). The 11 genes included four encoding interleukins (*IL-6, IL-8, IL-12B*, and *IL-10*), three encoding adhesion proteins (*CD40, VCAM*, and *ICAM1*), and four encoding chemokines (*Eotaxin-2, MPIF-1, MDC*, and *MCP-1*) (Fig. 2E).

We also examined the mRNA levels of the *TNF-α, MCP-1*, and *IL-1β* genes in NB4 cells with different levels of TG2 expression (NB4 WT, TG2-C, TG2-KD, and TG2-KO cells). TG2 expression strongly increased the mRNA expression of the *TNF-α, MCP-1*, and *IL-1β* genes in ATRA-treated cells, but this expression decreased in a dose-dependent manner with increasing 25(OH)D_3_ concentration (Fig. 2F). In general, TG2 expression correlated with an increase in the mRNA levels of proinflammatory proteins, which was suppressed by coadministration of 25(OH)D_3_.

### The cytokine storm induced by ATRA in differentiated NB4 WT cells is silenced in vitro by 25(OH)D_3_

**Fig. 3:**
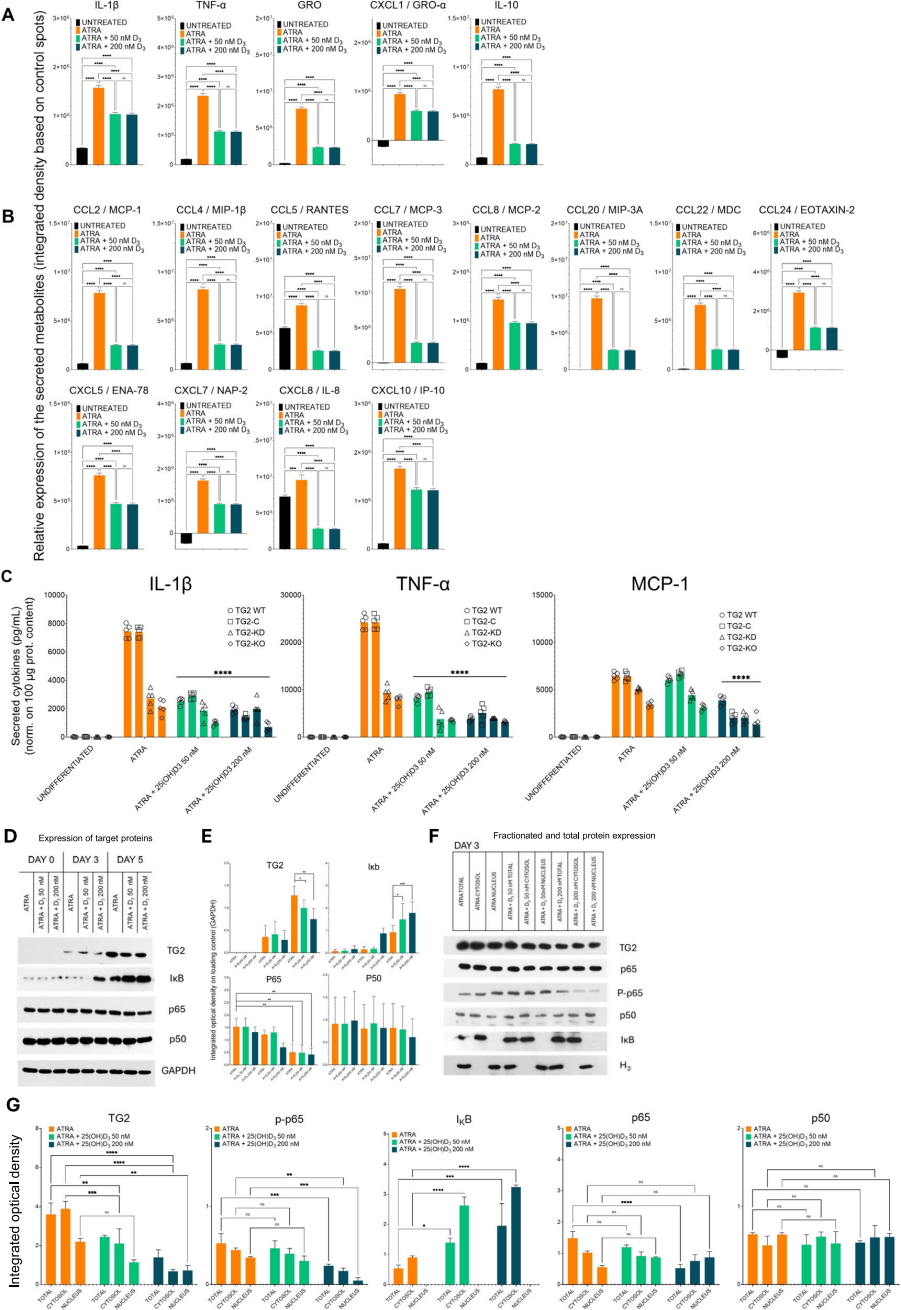
25(OH)D_3_ silences the cytokine storm induced by ATRA in differentiated NB4 cells in vitro. NB4 cells were treated for five days and harvested. Clear supernatants, free of cell debris, were used to measure secreted inflammatory analytes. Cytokine and chemokine levels, including MCP-1, TNF-α, and IL-1β, were measured using the ELISA MAX™ Deluxe Set in MAXISORB™ 96-well ELISA plates (Thermo Scientific). The relative expression of cytokines and chemokines was further evaluated using the ELISA-PLEX method (Abcam). **A, B** NB4 WT cells were treated with 1 µM ATRA, 1 µM ATRA + 50 nM 25(OH)D_3_, or 1 µM ATRA + 200 nM 25(OH)D_3_ for 5 days. Supernatants were collected and analysed using the ELISA-PLEX method to assess inflammatory cytokines and chemokines (*n* = 3), following the manufacturer’s instructions and recommended calculation methods. **C** NB4 cell lines were treated for five days, and the supernatants were collected and analysed by sandwich ELISA. The cytokine levels were normalized to protein concentrations. The graphs show the mean secreted cytokine values ± S.D. (*n* = 3). **D** Representative western blot showing TG2 and NF-κB pathway protein expression levels in total cell lysates after three and five days of treatment (*n* = 9). **E** Densitometry analysis shows the integrated optical density of total cell lysate samples. **F** Representative western blot showing TG2 and NF-κB pathway protein expression levels in total, cytosolic, and nuclear fractions after 3 and 5 days of treatment (*n* = 9). **G** Densitometry analysis shows the integrated optical density of the fractionated cell lysate samples. Statistical significance was determined using two-way analysis of variance (ANOVA) with Bonferroni post hoc test (*p < 0.05, **p < 0.01, ***p < 0.001, ****p < 0.0001).

Given that ATRA-differentiated NB4 cells produce the proinflammatory cytokines TNF-α and IL-1β at levels depend on TG2 expression, we used Abcam® cytokine arrays to identify several proinflammatory chemokines, as these proinflammatory cytokines stimulate the production of many chemokines, such as CXCL8 (IL-8), CCL2 (MCP-1), CCL3 (MIP-1α), CCL4 (MIP-1β), CCL5 (RANTES), and CXCL10 (IP-10). These chemokines are crucial for the inflammatory response because they attract immune cells to sites of inflammation. The administration of 25(OH)D3 to ATRA-treated cells affected the concentrations of proinflammatory cytokines, including IL-1β, TNF-α, GRO Pan, GRO-α, and IL-10, in the supernatant (Fig. 3A). Similarly, 25(OH)D_3_ cotreatment significantly reduced the expression of chemokines, such as CCL2/MCP-1, CCL4/MIP-1β, CCL5/RANTES, CCL7/MCP3, CXCL8/IL-8, CCL20/MIP-3A, CCL22/MDC and CCL24/EOTAXIN-2, CXCL5/ENA-78, CXCL7/NAP-2, CXCL8/IL-8 and CXCL10/IP10 (Fig. 3B). (Patients with DS have high plasma levels of CCL2/MCP-2, CCL4/MIP-1β, CCL7/MCP-3, CCL20/MIP-3α, CCL22/MDC, and CCL24/EOTAXIN-2.) ATRA-treated NB4 cells extensively secreted these chemokines, but this secretion, as well as the secretion of other NF-κB-regulated chemokines, was inhibited by more than two-thirds following cotreatment with 25(OH)D_3_ (Fig. 3B).

ELISA assessments of the concentrations of IL-1β, TNF-α, and MCP-1 confirmed that ATRA treatment increased the production of these molecules, and this increase was dependent on TG2 expression and was suppressed by cotreatment with 25(OH)D_3_ (Fig. 3C). Further western blot examinations of the total, cytosolic, and nuclear protein fractions containing TG2, p65, phospho-65, p50, and IκB confirmed that TG2 expression was reduced by day 5 of cotreatment with ATRA and 25(OH)D_3_. Another significant finding was that 25(OH)D_3_ markedly elevated the protein level of IκB (Fig. 3D, E). The compartmental (cytosolic, nuclear, and total) distributions of proteins on day 3 revealed a substantial decrease in TG2 and phospho-p65, which was associated with a significant increase in IκB (Fig. 3F). The western blot results for each target protein at day 5 (*n* = 9) indicate decreased TG2 and phospho-p65 levels, along with elevated IκB levels, after cotreatment with ATRA and 25(OH)D_3_ (Fig. 3G), suggesting that cotreatment contributes to suppressing the cytokine storm.

### Paricalcitol fails to attenuate ATRA-induced NF-κB activation significantly and cytokine release in differentiated NB4 cells compared to 25(OH)D_3_

Paricalcitol, a synthetic vitamin D_3_ analog, is clinically used to treat secondary hyperparathyroidism in chronic kidney disease. It exhibits potent anti-inflammatory effects by activating the vitamin D receptor (VDR), inhibiting NF-κB activity, and suppressing pro-inflammatory cytokines in chronic kidney disease or renal inflammation. The effects of two clinically used drugs on cell proliferation, NF-κB activation, and inflammatory cytokine inhibition were compared. The results are presented in the Supplementary Material.

### 25(OH)D_3_ inhibits cytokine production in healthy human donor neutrophil granulocytes after ex vivo induction by TNF-α

**Fig. 4:**
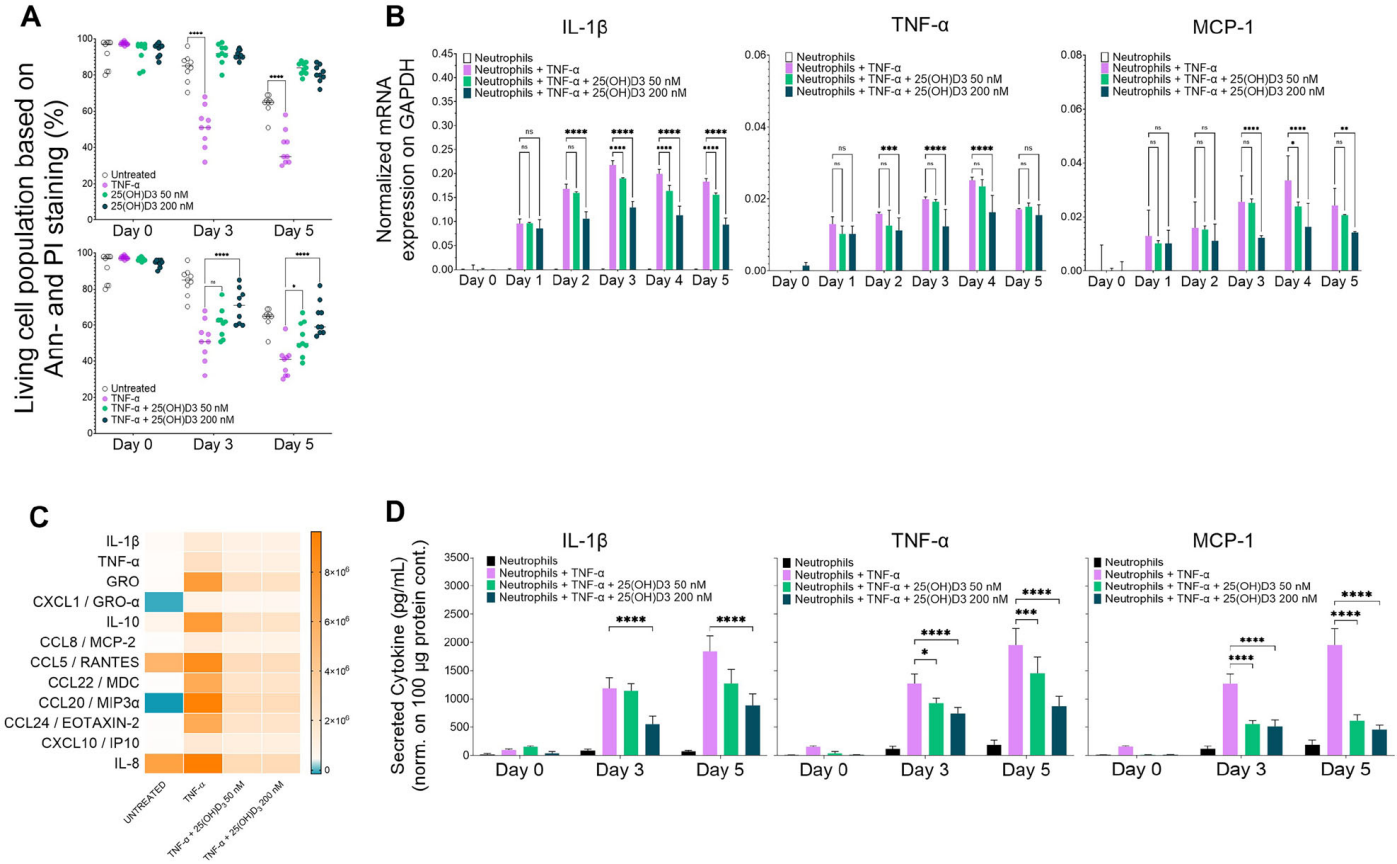
The TNF-α-induced cytokine storm in healthy human neutrophil granulocytes is silenced in vitro by 25(OH)D_3_. Healthy human neutrophil granulocytes were isolated from blood using the FICOLL method, followed by incubation with TNF-α, TNF-α + 50 nM 25(OH)D_3_, or TNF-α + 200 nM 25(OH)D_3_ for 5 days. **A** Cell viability of isolated neutrophils was assessed using Annexin-V and propidium iodide (P.I.) staining, followed by flow cytometry analysis (Flowing Software 1.02). The percentage of living cells was calculated, and the graph shows the mean values ± S.D. measured in triplicate on days 3 and 5 of treatment (*n* = 5). **B** Treated cells were harvested, and total RNA was isolated using the TRIzol method. cDNA synthesized from the resulting RT-PCR was used for RT-qPCR analysis. The graphs show relative mRNA expression levels of IL-1β, TNF-α, and MCP-1, calculated using the ^ΔΔ^Ct method (*n* = 5). **C** Supernatants were collected at the indicated time points from the same samples and analysed using ELISA PLEX (Abcam) (*n* = 3). The data was processed according to the manufacturer’s instructions using the recommended equations. **D** To examine cytokine and chemokine levels, IL-1β, TNF-α, and MCP-1 were measured using the ELISA MAX™ Deluxe Set on MAXISORB 96-well ELISA plates (Thermo Scientific). Values were normalized to the samples’ protein concentrations. Graphs show the mean values of secreted cytokines ± S.D. (*n* = 3). Statistical significance was determined via two-way analysis of variance (two-way ANOVA; Bonferroni post hoc test; *p < 0.05, **p < 0.01, ***p < 0.001, ****p < 0.0001).

The half-life of human neutrophils is uncertain, with estimates ranging from 7–9 h to 3.75 days. In this study, we found that the ex vivo apoptosis rate of neutrophils isolated from healthy human donors was approximately 35% on day 5. In ex vivo cell cultures, treatment with 25(OH)D_3_ at 50 and 200 nM improved neutrophil granulocyte viability, with 80% of the cells remaining alive after 5 days (Fig. 4A, left panel). Subsequent exposure of neutrophil granulocytes to TNF-α to simulate an inflammatory response reduced neutrophil viability to approximately 28–58%, with a mean of 38%, after 5 days. However, treatment with 25(OH)D_3_ significantly improved neutrophil survival, as 50% and 60% of the cells remained viable after treatment with 50 and 200 nM 25(OH)D_3_, respectively (Fig. 4A, right panel).

Measurement of TNF-α-induced inflammatory cytokine production (IL-1β, TNF-α, and MCP-1) at both the mRNA and secreted protein levels revealed that treatment with 50 and 200 nM 25(OH)D_3_ significantly reduced mRNA and protein expression by at least 50% on day 5 compared to TNF-α treatment alone (Fig. 4B). Abcam® cytokine arrays confirmed that both concentrations of 25(OH)D_3_ significantly reduced the levels of TNF-α–induced inflammatory cytokines, including IL-1β, TNF-α, GRO Pan, GRO-α, and IL-10 (Fig. 4C, upper row), as well as the chemokines CCL2/MCP-1, CCL5/RANTES, CCL8/MCP-2, CCL20/MIP-3α, CCL24/EOTAXIN-2, CXCL8/IL-8, and CXCL10/IP-10 (Fig. 4C, lower row). The accuracy of these measurements was corroborated by ELISA. Adding 50 or 200 nM 25(OH)D_3_ to ex vivo cultures reduced the levels of IL-1β, TNF-α, and MCP-1 (Fig. 4D), indicating that 25(OH)D_3_ can inhibit cytokine production in neutrophil granulocytes from healthy human donors following ex vivo induction by TNF-α.

### 25(OH)D_3_ has an anti-inflammatory effect on ATRA-differentiated ex vivo cell cultures from a patient with APL

**Fig. 5:**
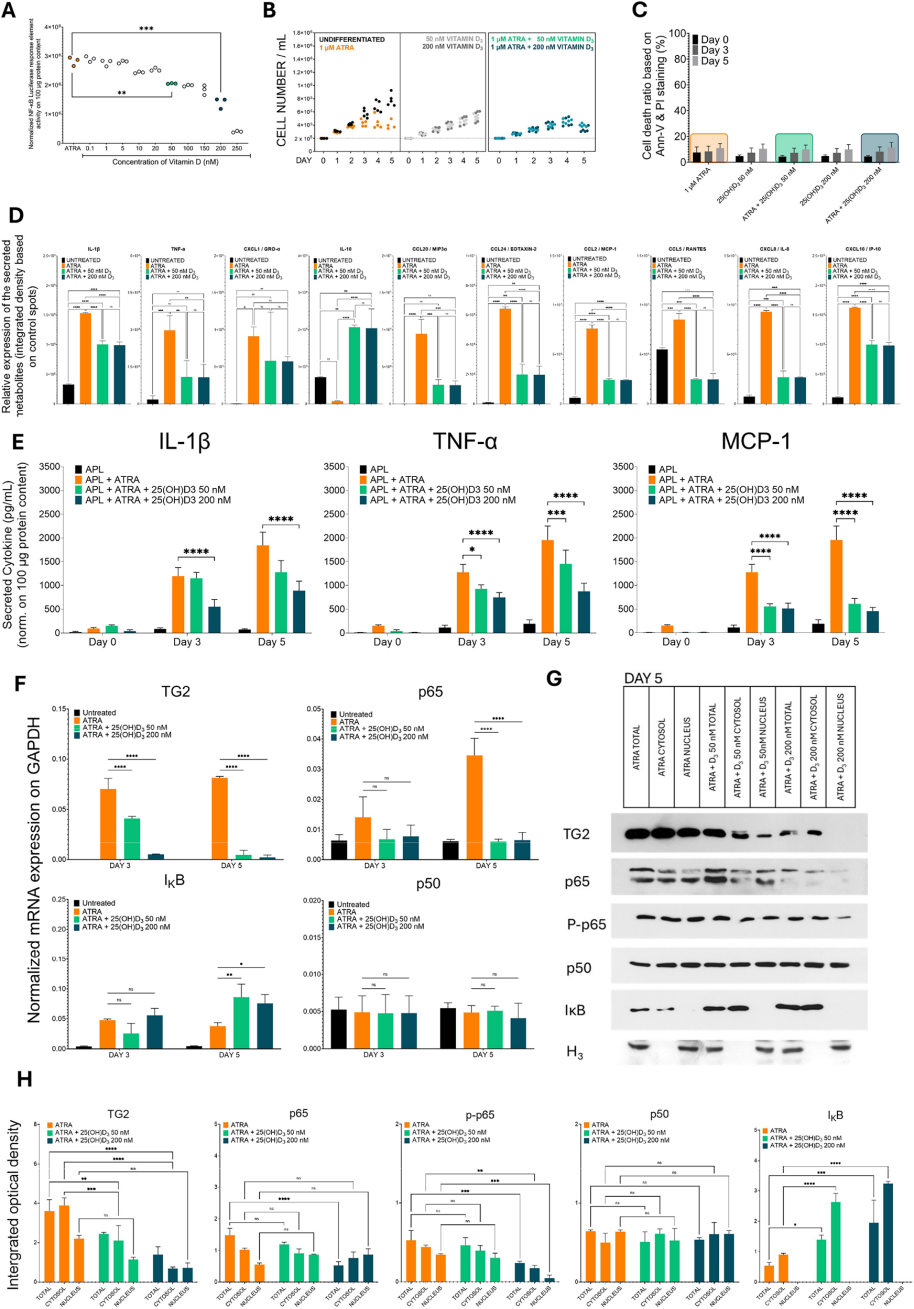
25(OH)D_3_ administration reduces NF-κB pathway activity in ATRA-differentiated cells from a patient with APL in ex vivo cultures. **A** APL cells were isolated from a patient using the FICOLL method, followed by transient transfection with an NF-κB response element-driven luciferase construct. Cells were treated with 1 µM ATRA or 1 µM ATRA plus increasing concentrations of 25(OH)D_3_. Luciferase reporter activity was measured in triplicate, and the relative light units (RLU) of treated and harvested cells are presented as mean ± S.D. (*n* = 5). **B** Cell numbers in the treated cultures were determined using KOVA™ Glasstic™ cell counting slides. The graphs show the number of cells measured in triplicate (mean ± S.D.) over five days (*n* = 5). **C** Cell viability was assessed using Annexin-V and propidium iodide staining, followed by flow cytometry analysis (Flowing software 1.02). The graph shows the mean percentage of viable cells ± S.D., measured in triplicate on day 5 (*n* = 5). **D** Culture supernatants from treated and harvested APL cells were collected for ELISA-PLEX analysis (*n* = 3), following the manufacturer’s instructions and recommended calculations. The graph shows the mean values of the relative levels of secreted analytes ± S.D. **E** Cytokine and chemokine levels, including IL-1β, TNF-α, and MCP-1 were measured using the ELISA MAX™ Deluxe Set on MAXISORB™ 96-well ELISA plates (Thermo Scientific). Cytokine levels were normalized to protein concentrations. The graphs present the mean values of secreted cytokines ± S.D. (*n* = 3). **F** Treated cells were harvested, and total RNA was isolated using the TRIzol method. The resulting cDNA from RT–PCR was used for RT–qPCR. The graphs show the relative mRNA expression of APL cells treated for 5 days, validated using the relative ^ΔΔ^Ct method (n = 5). **G** Representative western blot showing TG2 and NF-κB pathway protein expression levels in APL cells after 5 days of treatment in total, nuclear, and cytosolic fractions (*n* = 9). **H** Densitometry analysis results are presented as integrated optical density values normalized to the loading control. Statistical significance was determined using two-way analysis of variance (ANOVA) with Bonferroni post hoc test (*p < 0.05, **p < 0.01, ***p < 0.001, ****p < 0.0001).

A luciferase constructs stably integrated into the genomic DNA of permanent t(15;17) cells, derived from the leukemic cells of a patient with APL, confirmed that 1 µM ATRA increases NF-κB-mediated transcriptional activity in APL cells. Treatment with ATRA alone significantly increased NF-κB luciferase reporter gene activity, whereas cotreatment with up to 0.25 µM 25(OH)D_3_ significantly and dose-dependently reduced luciferase reporter gene activity by more than 90%. The addition of 50 and 200 nM 25(OH)D_3_ reduced luciferase reporter gene activity by 33% and over 50%, respectively (Fig. 5A).

Cultures treated with 50 or 200 nM 25(OH)D_3_ showed no significant differences in cell counts, regardless of the presence or absence of ATRA (Fig. 5B). Apoptosis assessments of APL cells using annexin-V and propidium iodide double labeling revealed no significant differences in the cell death ratio following treatment with 50 or 200 nM 25(OH)D_3_, whether ATRA was present (Fig. 5C).

ATRA-induced cytokines, including many of those commonly elevated in the plasma of severely ill patients with DS or intracranial hemorrhage (ICH), were examined via a human cytokine protein array in ex vivo APL cultures. The levels of IL-1β, TNF-α, CXCL1/GRO-α, IL-10, CCL2/MCP-1, CCL5/RANTES, CXCL8/IL-8, CXCL10/IP-10, CCL20/MIP-3α, and CCL24/EOTAXIN-2 were significantly increased in the supernatants of ATRA-treated APL cells after 5 days (Fig. 5D). Ex vivo APL cultures cotreated with ATRA and either 50 or 200 nM 25(OH)D_3_ exhibited significantly lower cytokine levels (Fig. 5D, light and dark blue columns). High-specificity ELISA confirmed that cotreatment with ATRA and 25(OH)D_3_ reduced the levels of IL-1β, TNF-α, and MCP-1 by more than 50% to 75% (Fig. 5E).

RT-qPCR analysis revealed that TG2 and p65 expression significantly decreased in response to cotreatment with 25(OH)D_3_, while IκB expression increased, and p50 levels remained unchanged (Fig. 5F). Immunoblotting experiments showed significantly lower nuclear levels of TG2 and phospho-p65 proteins, along with significantly higher levels of IκB protein, in APL cells after 5 days of cotreatment with ATRA and 25(OH)D_3_ (Fig. 5G–H). These findings suggest that cytokine levels decreased following 25(OH)D_3_ therapy because the NF-κB complex became retained in the cytosol at reduced levels.

## Discussion

Transglutaminase 2 has emerged as a new and essential survival factor in a variety of cancer cell types. TG2, a GTP/GDP-binding protein, hydrolyzes GTP and functions as a G protein and it is now recognized as a key cancer cell survival factor that interacts with signaling pathways that drive vital cancer-related functions, including drug resistance, cancer stem cell survival, metastasis, inflammation, epithelial‒mesenchymal transition, and angiogenesis [16, 17].

The findings presented here indicate that the suppression of NF-κB expression and translocation may be an effective treatment strategy for APL. The NF-κB protein complex has an essential function in controlling the immunological response to infection in normal, healthy cells. A complex connection exists between NF-κB expression and cancer, encompassing its role in inflammation, cell survival, apoptosis, proliferation, angiogenesis, and metastasis [20–22,18, 23–25]. The activation of NF-κB may be triggered by genetic changes in the NF-κB pathway or by chronic inflammation, as observed in atypically expressed TG2. NF-κB is a potential cancer therapeutic candidate because of its significant role in cancer biology and in the development of DS.

In the case of APL, ATRA therapy can be used to differentiate human APL cells into neutrophil granulocytes. However, approximately 30% of patients experience DS, with death occurring in 1–5% of the affected patients. A new study indicated that failure to promptly identify intracranial hemorrhage (ICH) in patients with APL may result in fatal outcomes. In this case, the cytokine storm was deemed the primary cause of induction therapy failure and early mortality [7]. Further examination of constitutive abnormalities in APL patient plasma identified translatable biomarkers for APL-related ICH [7]. Comparison of preliminary plasma cytokine and chemokine profiles from 39 APL patients and 18 healthy donors revealed significant differences in Eotaxin, GROα, IL-1α, IL-1β, IL-10, IL-15, IL-1Ra, IL-2Ra, IL-5, IL-6, IL-8, IP-10, M-CSF, MCP-1, MIP-1β, RANTES, SCGFb, SDF-1a, and TNF-α. In the present study, cell cultures from both a leukemic APL patient and NB4 cells had lower levels of cytokines (marked in bold) when they were cotreated with ATRA and 25(OH)D_3_, which may suggest that the administration of 25(OH)D_3_ can also be preventive in APL patients.

Our study tested the effects of both ATRA alone and in combination with up to 250 nM 25(OH)D_3_ on luciferase reporter gene activity in leukemic cells from a patient with APL. ATRA alone significantly enhanced NF-κB promoter-driven luciferase reporter gene activity, but this activity was dose-dependently decreased by more than 90% by cotreatment with up to 250 nM 25(OH)D_3_. Cotreatment with 25(OH)D_3_ in ATRA-treated NB4 cell lines led to a gradual decline in NF-κB promoter activity, which was in turn directly correlated with the expression levels of TG2 in NB4 WT, NB4 TG2-C, and NB4 TG2-KD cells. However, in the absence of TG2 expression, 25(OH)D_3_ no longer affected NF-κB promoter activity, as was evident in NB4 TG2-KO cells (white column) (Fig. 1D). Cotreatment with 25(OH)D_3_ also diminished the transcription of NF-κB-related genes (*REL, RELB, NFkB1*, and *NFkB2*) in ATRA-differentiated NB4 WT cells while increasing the expression of the transcription-inhibitory genes *NFkBIA, NFkBIE*, and *NFkBIB*, which is consistent with the observed reduction in the expression of proinflammatory cytokines and chemokines (Fig. 2A–E).

For three decades, the production of proinflammatory cytokines, such as IL-1β, IL-6, IL-8, and TNF-α, by leukemic cells in ATRA-differentiated APL patients has been recognized to result in DS and subsequent systemic inflammatory response syndrome (SIRS) [26]. Additional chemokines, such as CCL1/II-309, CCL2/MCP-2, CCL4/MIP-α, CCL7/MCP-3, CCL20/MIP-3α, CCL22/MDC, CCL24/EOTAXIN-2, IL-1α, IL-15, IL-1Ra, IL-2Ra, IL-5, IL-6, M-CSF, SCGFb, and SDF-1a, have since been detected in the plasma of DS patients [7, 27]. Vitamin D_3_ supplementation can significantly reduce TNF-α levels in the bloodstream of cancer patients [28]. Our combined treatment with ATRA and 25(OH)D_3_ reduced cytokine levels by one-third to three-quarters in more than 50% of our samples (Fig. 3A, B). The changes in the concentrations of IL-1β, TNF-α, and MCP-1 in NB4 WT, TG2-C, TG2-KD, and TG2-KO cell cultures were confirmed via ELISA, which revealed strong correlations between TG2 levels and the concentrations of IL-1β, TNF-α, and MCP-1 in the supernatant. The use of higher 25(OH)D_3_ levels in the combined treatment significantly reduced these concentrations, particularly in the NB4 WT, NB4-C, and NB4 TG2-KD cells that expressed TG2. The NB4 TG2-KO cells, which completely lacked TG2, exhibited the lowest concentration of cytokines and showed only a slight decrease in response to 25(OH)D_3_ (Fig. 3C).

TNFR and Fas are known to initiate apoptosis in neutrophils, but TNF receptors induce survival mechanisms in human neutrophils [29] by activating the p38-MAPK pathway, which leads to the release of the antiapoptotic chemokine IL-8 [30, 31]. To validate the NB4 cell culture results, we examined the effects of TNF-α and 25(OH)D3 on ex vivo isolated neutrophil granulocytes. This revealed increased cell death in response to TNF-α compared with spontaneous neutrophil granulocyte death in response to TNF-α compared with spontaneous neutrophil granulocyte death, whereas 25(OH)D_3_ reduced cell death.

The ex vivo combination of TNF-α and 25(OH)D3 generated a cytokine expression profile similar to that observed with ATRA and 25(OH)D3 in NB4 WT cells, suggesting that 25(OH)D3 may also suppress inflammatory cytokine expression in TNF-α-activated neutrophil granulocytes, potentially in vivo (Fig. 4D).

The overproduction of inflammatory mediators, including cytokines, directly results from ATRA-induced TG2 and TG2-mediated NF-κB activation [32, 33]. TNF-α, IL-1β, and MCP-1, among other cytokines, may be indicators of illness progression, mortality, or severity in DS or ICH [26, 34]. Corticosteroids are given to suppress inflammation in DS and ICH patients [27]. We found that 25(OH)D_3_, a natural steroid hormone, significantly inhibited the expression of TG2 and phospho-p65 proteins while increasing IκB-α protein levels, thereby reducing p65 translocation into the nucleus and preventing NF-κB transcriptional activation and activity. In the ATRA-treated cells from the APL patient, this markedly diminished the protein expression levels of TNF-α, IL-1β, and MCP-1, as well as the gene expression levels of *TG2* and *p65*, while enhancing *IκB*. These findings suggest that 25(OH)D_3_ may be a viable treatment for DS and ICH.

In conclusion, the results presented in this study reveal the significant anti-inflammatory benefit of 25(OH)D_3_, a natural steroid hormone, against the development of DS and possibly ICH in patients with APL by reducing NF-κB nuclear translocation and the subsequent cytokine storm. 25(OH)D3 can reduce leukemic cytokine levels by increasing IκB protein levels and decreasing phospho-p65 and TG2 protein levels. Our findings suggest that administering 25(OH)D_3_ as a supplementary therapy for treating APL could complement current ATRA therapy [35].

## Supplementary information

Original data

Supplementary methods and figure

Supplementary figures

## Data Availability

Reagents and data can be accessed as appropriate by contacting jambrovics.karoly@med.unideb.hu.
