# Supplementary material for: Anti-inflammatory 25(OH)D3, a natural steroid hormone, may complement all-trans retinoic acid therapy for differentiation syndrome in acute promyelocytic leukemia
Source: Cell Death Dis. 2025 Nov 3;16(1):787. doi: 10.1038/s41419-025-08109-7 (PMC12583587; doi:10.1038/s41419-025-08109-7)
Supplement: Supplementary file 1 — Original data [file 41419_2025_8109_MOESM1_ESM.pdf]

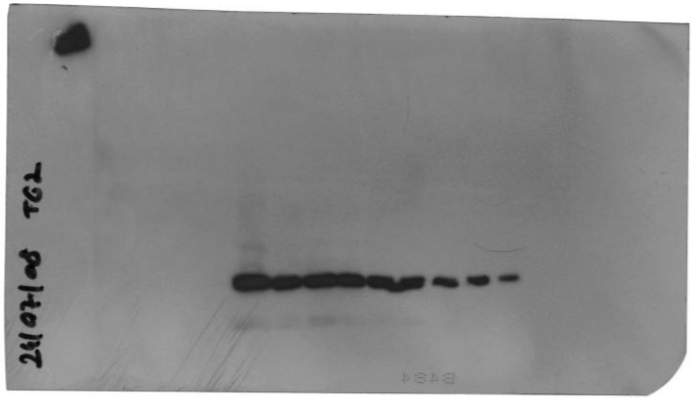
24/07/08 TG2

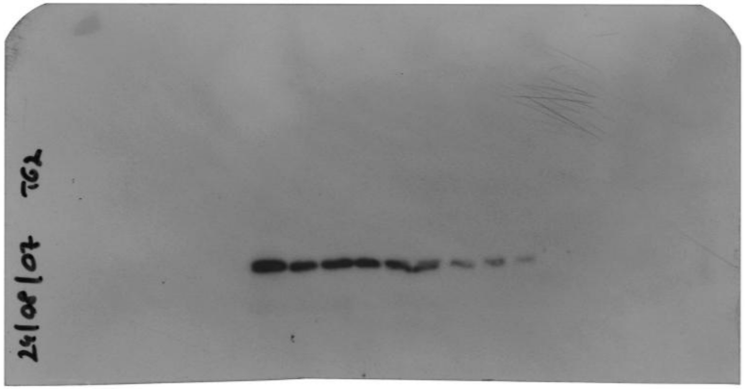
24/08/07 TG2

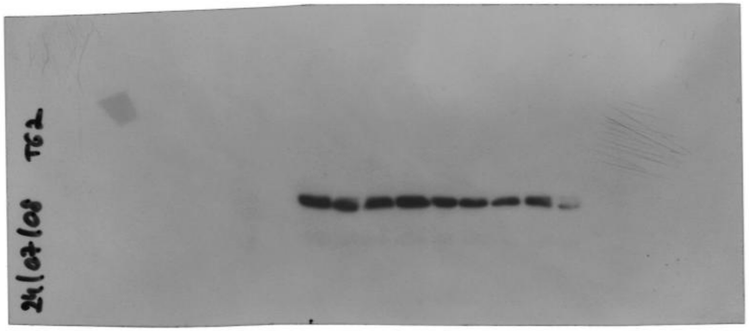
24/07/08 TG2

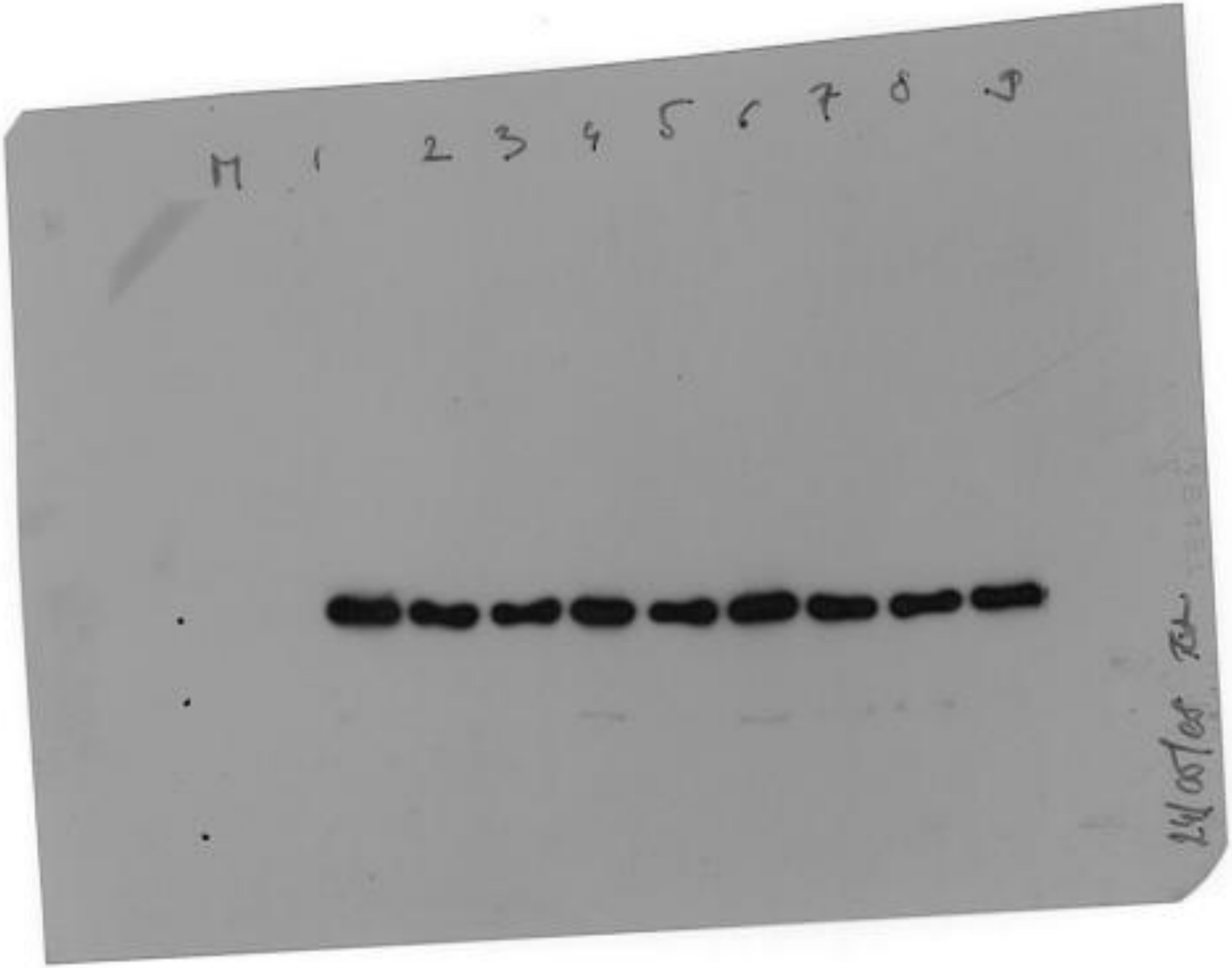
M 1 2 3 4 5 6 7 8 9

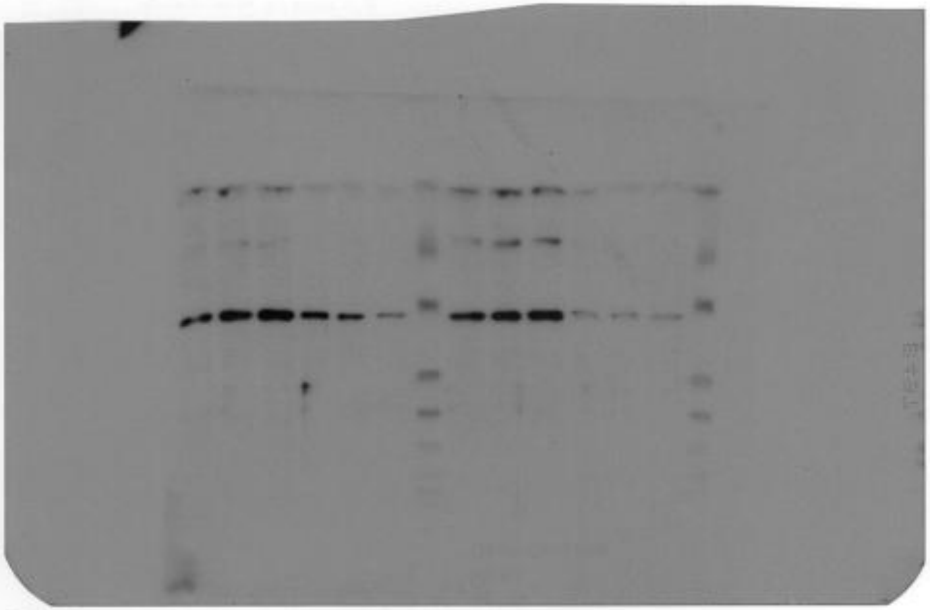

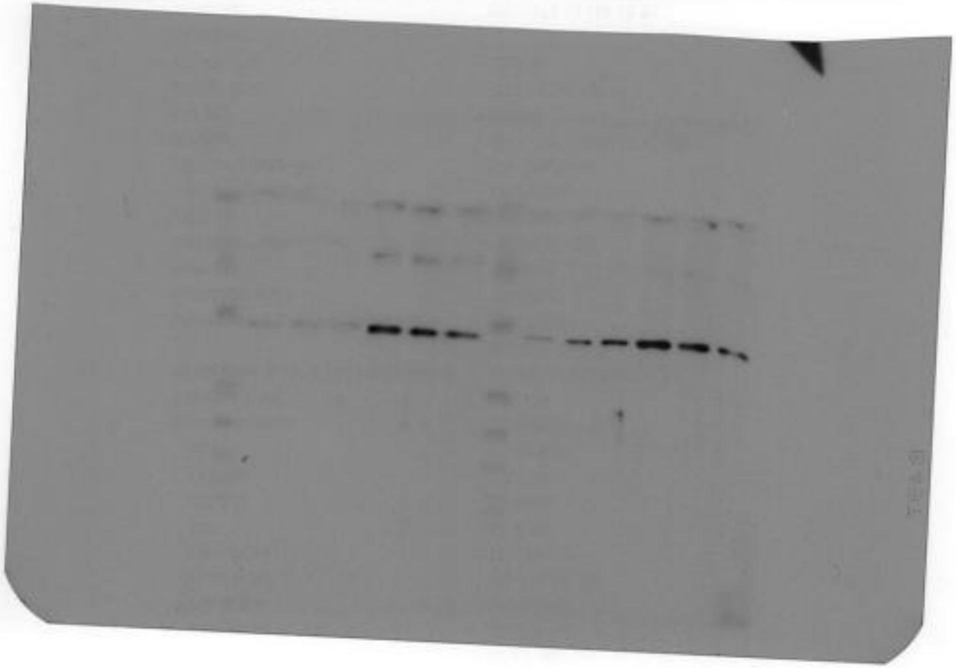

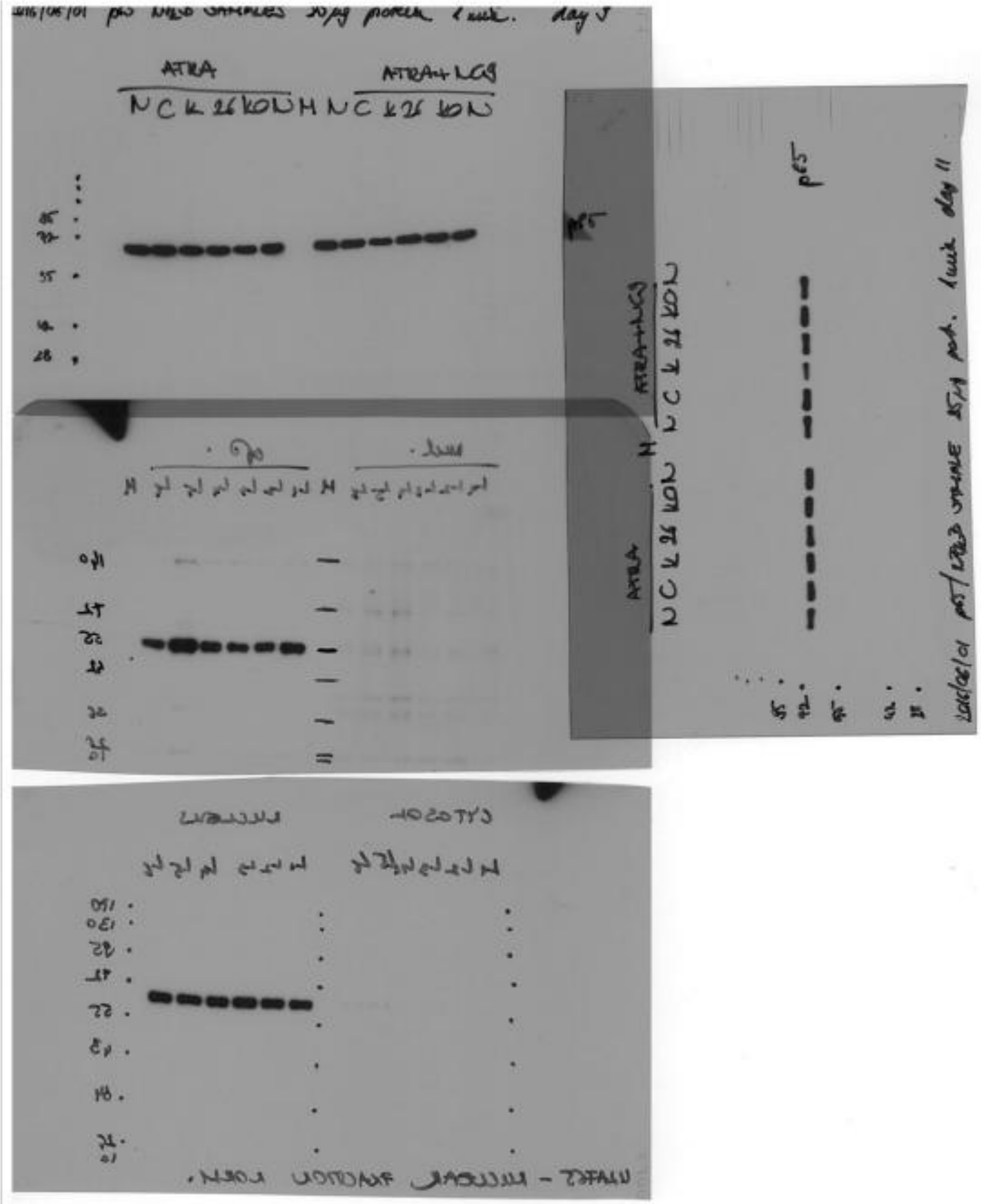
ATRA
p53

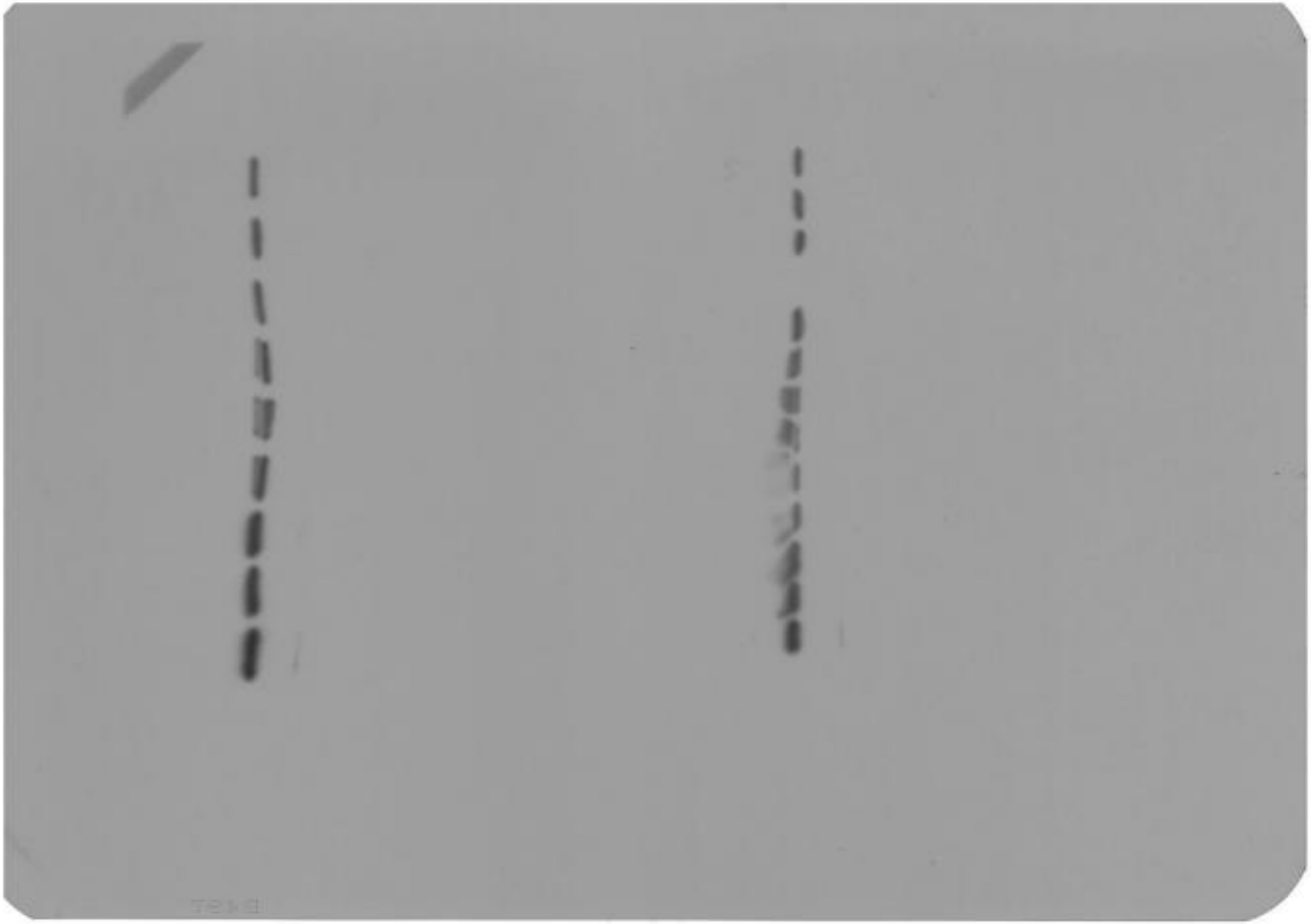

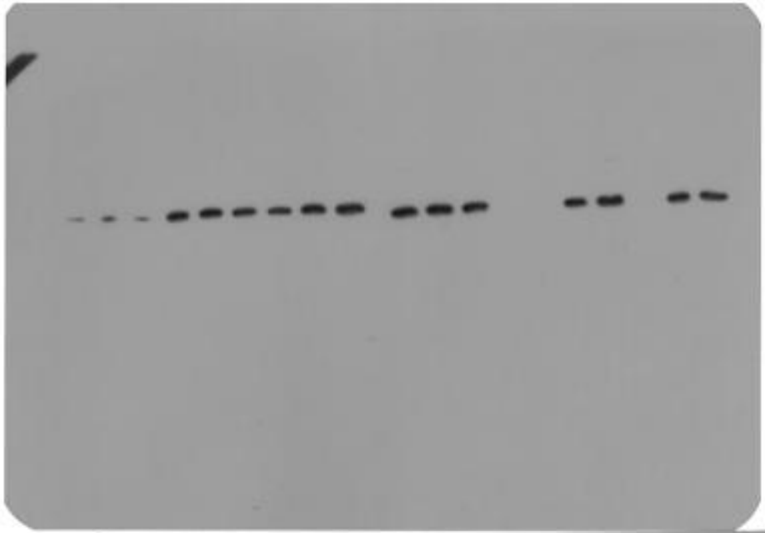
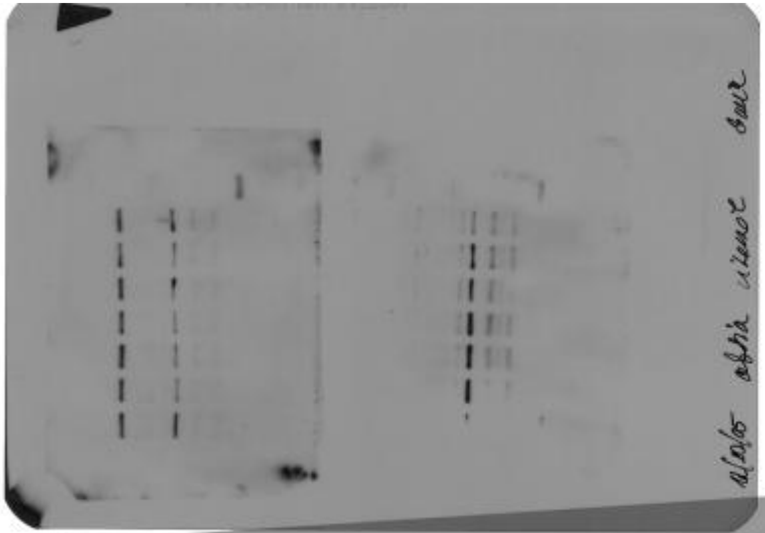
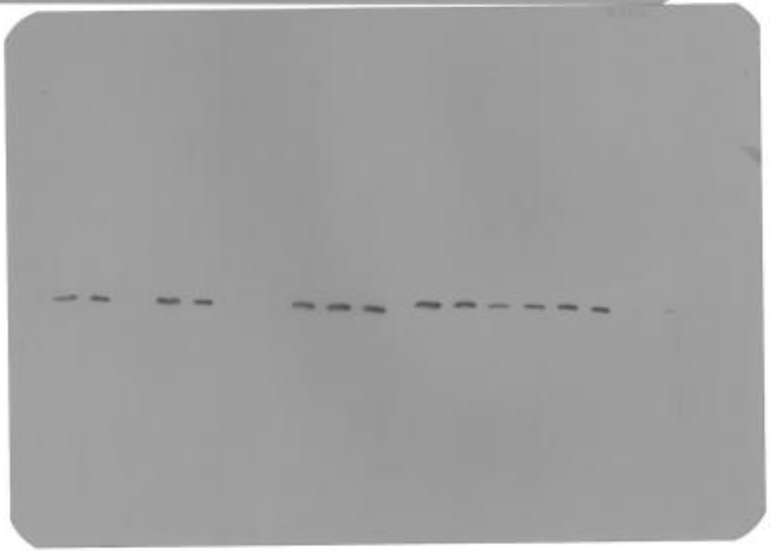
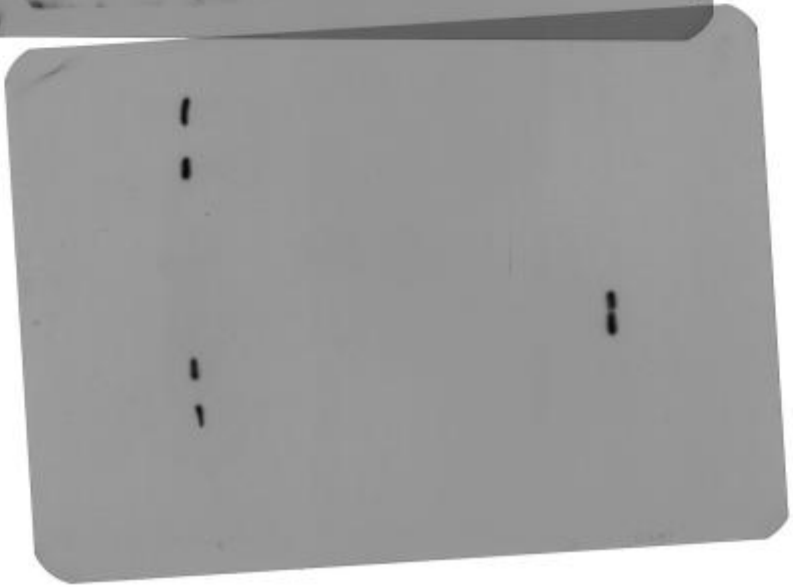

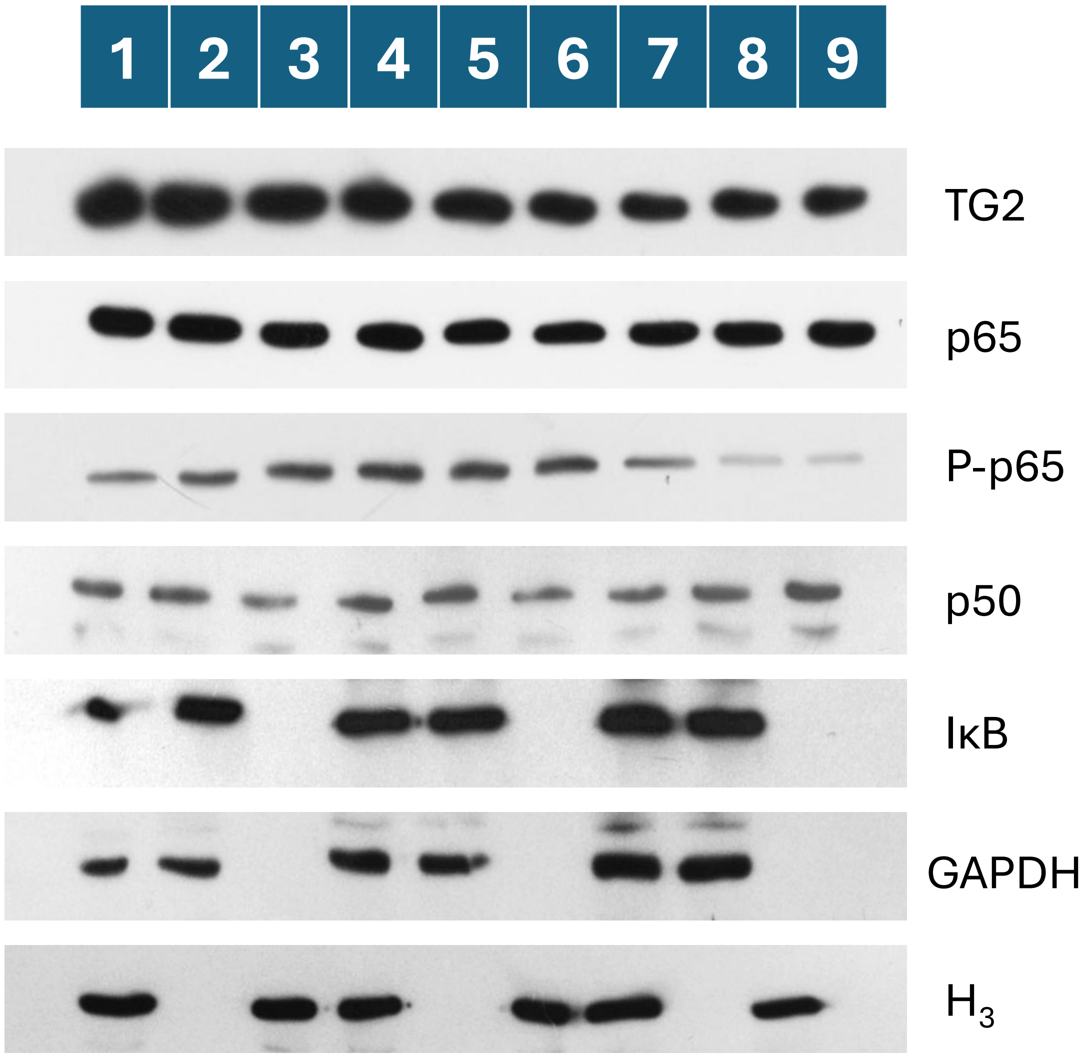

NB4 SAMPLES DAY3

1	ATRA TOTAL
2	ATRA CYTOSOL
3	ATRA NUCLEUS
4	ATRA + Vitamin D 50 TOTAL
5	ATRA + Vitamin D 50 CYTOSOL
6	ATRA + Vitamin D 50 NUCLEUS
7	ATRA + Vitamin D 200 TOTAL
8	ATRA + Vitamin D 200 CYTOSOL
9	ATRA + Vitamin D 200 NUCLEUS

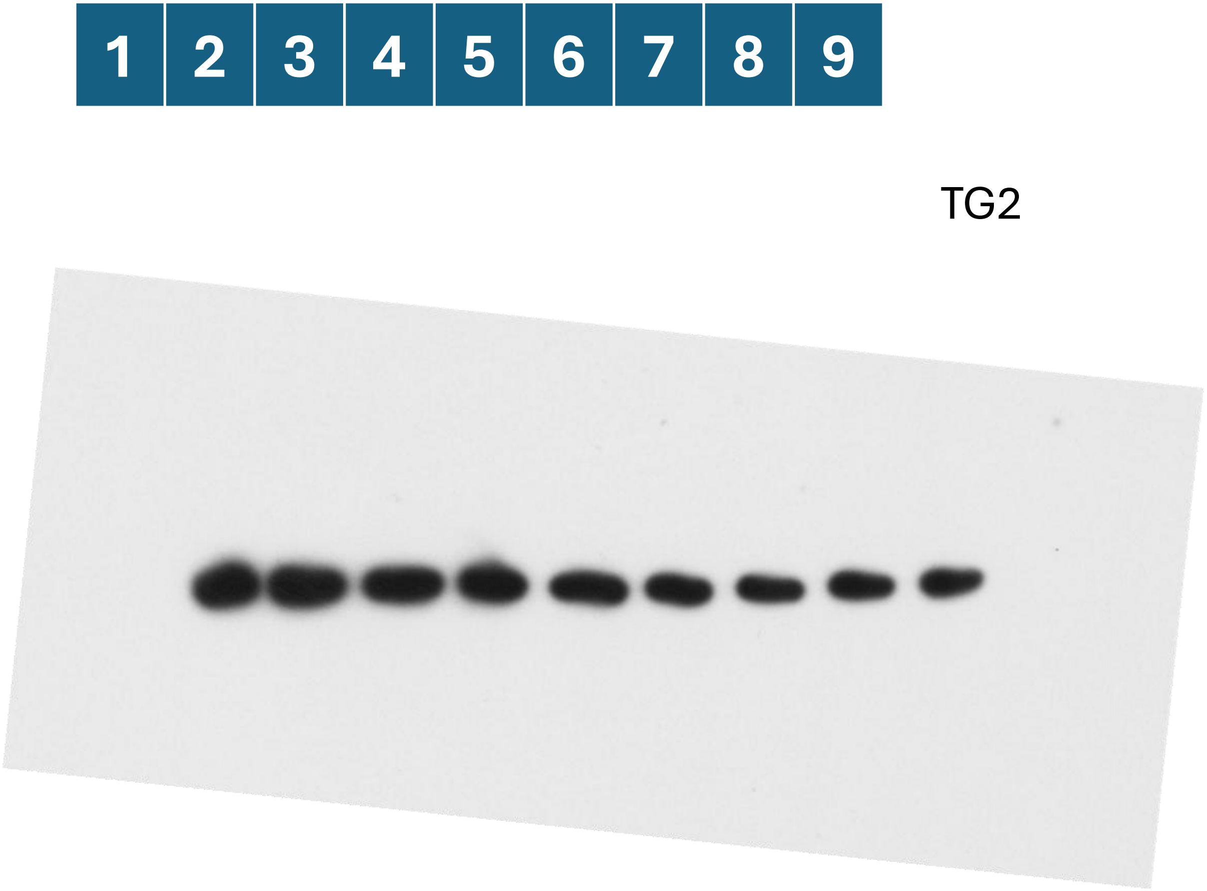

NB4 SAMPLES

1	**ATRA TOTAL**
2	ATRA CYTOSOL
3	ATRA NUCLEUS
4	ATRA + Vitamin D 50 TOTAL
5	ATRA + Vitamin D 50 CYTOSOL
6	ATRA + Vitamin D 50 NUCLEUS
7	ATRA + Vitamin D 200 TOTAL
8	ATRA + Vitamin D 200 CYTOSOL
9	ATRA + Vitamin D 200 NUCLEUS

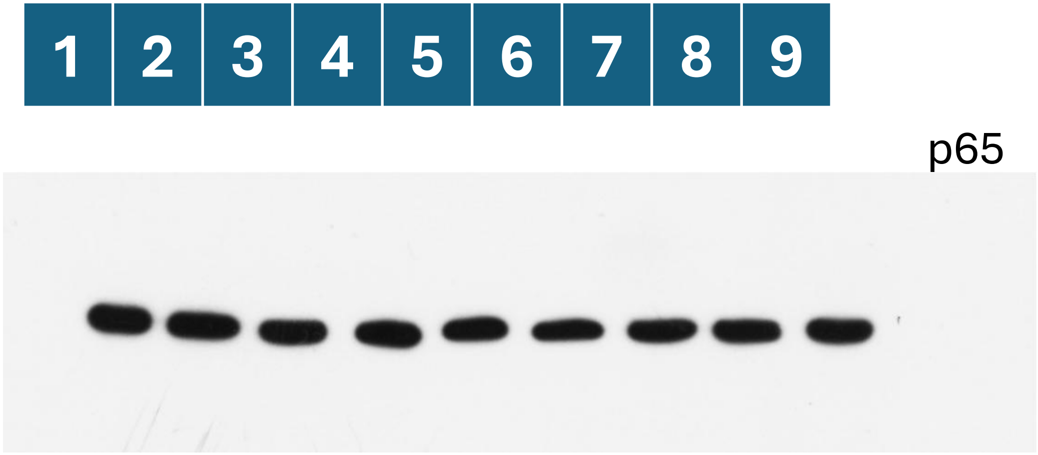

NB4 SAMPLES DAY

1	**ATRA TOTAL**
2	ATRA CYTOSOL
3	ATRA NUCLEUS
4	ATRA + Vitamin D 50 TOTAL
5	ATRA + Vitamin D 50 CYTOSOL
6	ATRA + Vitamin D 50 NUCLEUS
7	ATRA + Vitamin D 200 TOTAL
8	ATRA + Vitamin D 200 CYTOSOL
9	ATRA + Vitamin D 200 NUCLEUS

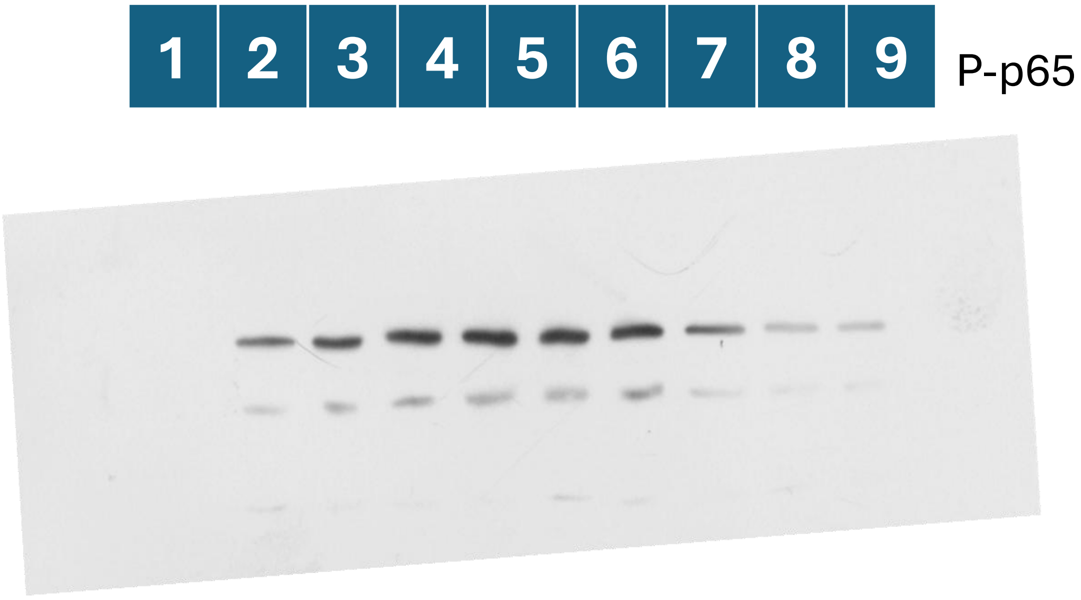

NB4 SAMPLES DAY3

1	ATRA TOTAL
2	ATRA CYTOSOL
3	ATRA NUCLEUS
4	ATRA + Vitamin D 50 TOTAL
5	ATRA + Vitamin D 50 CYTOSOL
6	ATRA + Vitamin D 50 NUCLEUS
7	ATRA + Vitamin D 200 TOTAL
8	ATRA + Vitamin D 200 CYTOSOL
9	ATRA + Vitamin D 200 NUCLEUS

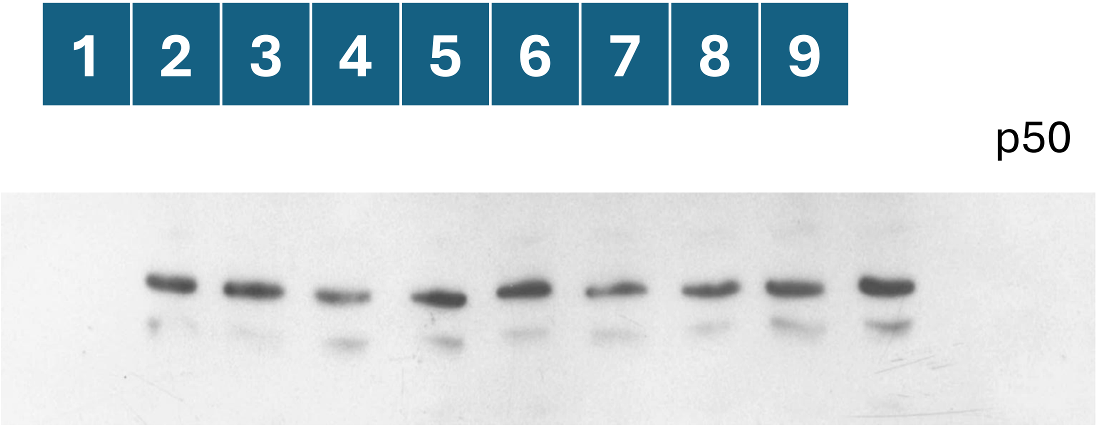

NB4 SAMPLES DAY3

1	**ATRA TOTAL**
2	ATRA CYTOSOL
3	ATRA NUCLEUS
4	ATRA + Vitamin D 50 TOTAL
5	ATRA + Vitamin D 50 CYTOSOL
6	ATRA + Vitamin D 50 NUCLEUS
7	ATRA + Vitamin D 200 TOTAL
8	ATRA + Vitamin D 200 CYTOSOL
9	ATRA + Vitamin D 200 NUCLEUS

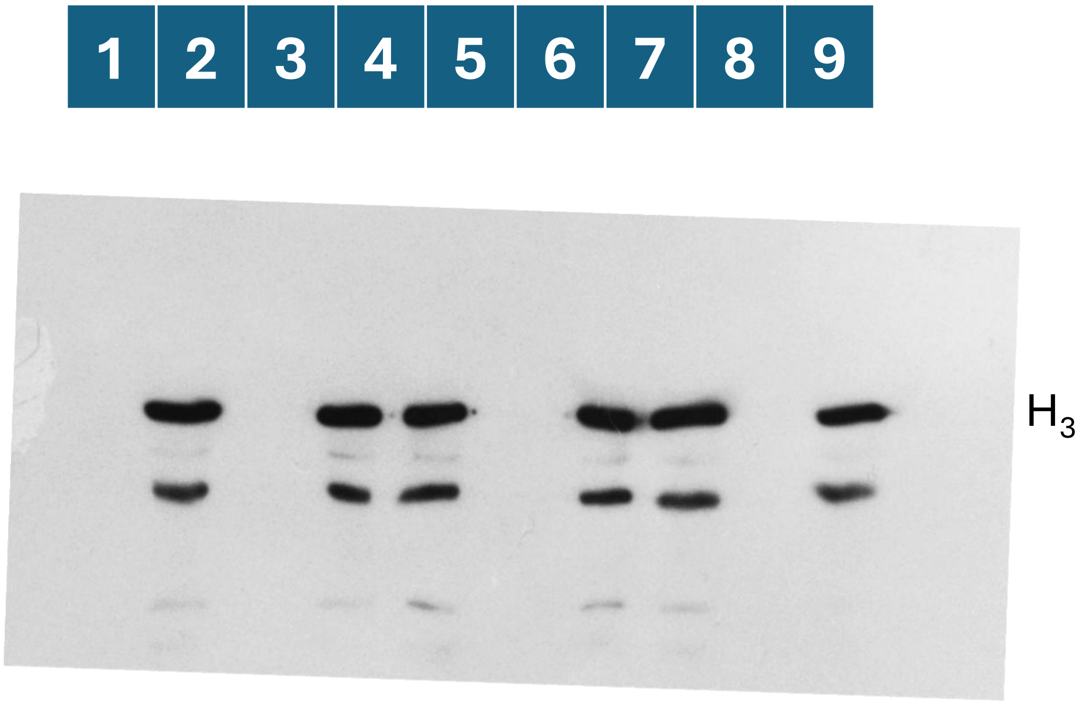

NB4 SAMPLES DAY3

1	ATRA TOTAL
2	ATRA CYTOSOL
3	ATRA NUCLEUS
4	ATRA + Vitamin D 50 TOTAL
5	ATRA + Vitamin D 50 CYTOSOL
6	ATRA + Vitamin D 50 NUCLEUS
7	ATRA + Vitamin D 200 TOTAL
8	ATRA + Vitamin D 200 CYTOSOL
9	ATRA + Vitamin D 200 NUCLEUS
