# Supplementary material for: Anti-inflammatory 25(OH)D3, a natural steroid hormone, may complement all-trans retinoic acid therapy for differentiation syndrome in acute promyelocytic leukemia
Source: Cell Death Dis. 2025 Nov 3;16(1):787. doi: 10.1038/s41419-025-08109-7 (PMC12583587; doi:10.1038/s41419-025-08109-7)
Supplement: Supplementary file 2 — Supplementary methods and figure [file 41419_2025_8109_MOESM2_ESM.docx]

**Supplementary Materials and Methods**

**Cell culture and treatments**

NB4 cell lines were cultured in RPMI-1640 medium (Merck KGaA, Darmstadt, Germany) supplemented with 10% (v/v) fetal bovine serum (FBS) (Gibco, Paisley, Scotland), 2 mM L-glutamine (Merck KGaA), 1% (v/v) 100 U/mL penicillin‒streptomycin solution, and 1% (v/v) sodium pyruvate solution (10 mg/mL) (Merck KGaA) at 37°C in a humidified atmosphere containing 5% CO₂. All cell lines were routinely tested for mycoplasma contamination (Promokine, Biomedica Hungaria Kft.) prior to experiments. For differentiation, NB4 cells were treated with 1 µM all-trans retinoic acid (ATRA) (Merck KGaA) or with various concentrations of 25-(OH)-D₃ (Merck KGaA) alone or in combination with ATRA for 3 or 5 days.

**Isolation and culture of human neutrophils from peripheral blood**

Peripheral blood was collected into EDTA-coated tubes to prevent coagulation and maintain neutrophils in suspension. The blood was diluted 1:1 with sterile phosphate-buffered saline (PBS) to reduce viscosity and facilitate cell separation. Dextran sedimentation using a 3% dextran solution was performed for 30–60 min at room temperature to promote erythrocyte (RBC) aggregation and sedimentation.

The leukocyte-rich supernatant was carefully aspirated and layered over Ficoll-Paque (density: 1.077 g/mL) to separate mononuclear cells from granulocytes based on their differential densities. Centrifugation was performed at 400 × g for 30 min at room temperature without braking. Distinct layers formed: plasma, a mononuclear cell layer, Ficoll-Paque, and a granulocyte‒RBC pellet.

The granulocyte-enriched fraction was collected and treated with ammonium chloride potassium (ACK) lysis buffer to selectively lyse RBCs. Lysis was performed on ice for 5–10 min to prevent neutrophil activation and was stopped by adding PBS. Neutrophils were pelleted by centrifugation at 300 × g for 10 min, washed twice with PBS, and resuspended in culture medium. Cell counts were determined using a hemocytometer or automated cell counter, and neutrophil purity (>90%) was confirmed via flow cytometry or light microscopy.

Neutrophils were cultured in RPMI-1640 supplemented with 10% heat-inactivated FBS and, when required, recombinant human granulocyte‒macrophage colony-stimulating factor (GM-CSF) or granulocyte colony-stimulating factor (G-CSF). Cells were seeded at 0.5–1 × 10⁶ cells/well (24-well plates) or 0.1–0.2 × 10⁶ cells/well (96-well plates) and incubated at 37°C in a humidified atmosphere containing 5% CO₂. For experiments exceeding 12 h, 50% of the medium was replaced with fresh, prewarmed medium containing GM-CSF or G-CSF to improve viability. This protocol preserved neutrophil viability and function for up to 2–5 days.

**Cell counting and evaluation**

Cell counts were performed using KOVA™ Glasstic™ cell counting slides according to the manufacturer's instructions.

**Transduction of NB4 cells and luciferase activity measurement**

NF-κB pathway activity in NB4 cells was measured using the QIAGEN CLS-013-L8 lentiviral NF-κB luciferase reporter system (QIAGEN), following the manufacturer’s instructions. Luciferase activity was measured using the Bright-Glo™ Luciferase Assay System (Promega, E2610) and normalized to total protein concentration.

**Flow cytometry**

Treated cell cultures (1 × 10⁵ cells in 200 µL) were centrifuged at 55 × g for 3 min at 4°C. The supernatant was discarded, and cells were resuspended in a cell death detection buffer containing propidium iodide (50 µg/mL) and FITC-labeled annexin-V (50 µg/mL). Samples were incubated in the dark for 15 min at 4°C and analyzed using a BD FACSCalibur flow cytometer with BD CellQuest Pro software. Data were analyzed using Flowing software version 2.13.

**Real-time quantitative PCR (RT‒qPCR)**

RT‒qPCR was performed using TaqMan™ probes (Applied Biosystems) for GAPDH, MCP-1, TNF-α, IL-1β, TG2, p65, p50, and IκB on a Roche LightCycler® 480 II system. The relative ^ΔΔ^Ct method was used for analysis.

**ELISA and ELISA-PLEX**

Cytokine and chemokine levels (MCP-1, TNF-α, and IL-1β) were measured using the ELISA MAX™ Deluxe Set (BioLegend) on MAXISORB™ 96-well ELISA plates (Thermo Fisher Scientific). ELISA-PLEX assays were performed using precoated multiplex membranes (Abcam) according to the manufacturer's protocols.

**TaqMan™ Array Human NF-κB Pathway**

The NF-κB pathway was profiled using the TaqMan™ Array Human NF-κB Pathway (Thermo Fisher Scientific, 4391139) following the manufacturer’s instructions. Total RNA was isolated, reverse transcribed, and analyzed via RT‒qPCR using the ΔΔCt method.

**SDS-PAGE and Western blotting**

A total of 1–2 × 10⁶ cells were lysed in buffer (50 mM Tris, 1 mM EDTA, 0.5% Triton X-100, 1 mM PMSF, and 0.1 M MEA) or fractionated into nuclear and cytosolic extracts (Merck KGaA) in the presence of protease inhibitor cocktail (PIC, 1:100 dilution). Cells were sonicated (Branson Sonifier® 450, 5–7 strokes, 40% cycle) and centrifuged at 13,700 × g for 15 min at 4°C. Nuclear and cytosolic fractionation was performed according to the manufacturer's instructions (Jambrovics et al., *Haematologica*).

Protein concentration was determined via Bradford assay (Bio-Rad) in triplicate using a Synergy™ multi-mode microplate reader (BioTek Instruments, Inc.) at 595 nm. Samples were diluted to 2 mg/mL, mixed with 2× SDS loading buffer (0.125 M Tris-HCl, pH 6.8, 4% SDS, 20% glycerol, 10% MEA, and 0.02% bromophenol blue), and denatured at 99°C for 10 min. Proteins were resolved by 8–10% SDS-PAGE, transferred to PVDF membranes (Merck KGaA), blocked with 5% non-fat dry milk in 1× TTBS for 1 h at room temperature, and incubated overnight at 4°C with primary antibodies diluted 1:1000–1:5000 in 0.5% milk/TTBS. After three 15 min washes, membranes were incubated for 1 h with HRP-conjugated secondary antibodies (Advansta, 1:10,000–1:20,000). Signals were detected using an enhanced chemiluminescence (ECL) kit (Advansta) and quantified using ImageJ software version 1.12.

**Statistical analysis**

Statistical analysis was performed using GraphPad Prism version 9.1.8. Two-way ANOVA with Bonferroni post hoc multiple comparisons was used. Statistical significance was set at *p* < 0.05, **p** < 0.01, ***p*** < 0.001.

**Paricalcitol: a clinical vitamin D₃ analogue**

Paricalcitol is a vitamin D₃ analogue used clinically, primarily in the treatment of secondary hyperparathyroidism in patients with chronic kidney disease. Substantial evidence from molecular studies, animal models, and clinical trials in chronic kidney disease, dialysis, and transplant populations indicates that paricalcitol exerts potent anti-inflammatory effects. These effects are mediated through direct activation of the vitamin D receptor (VDR), inhibition of NF‑κB/p65 transcriptional activity, and suppression of key pro-inflammatory cytokines^1-3^.

In addition to its anti-inflammatory properties, paricalcitol has demonstrated anti-leukemic activity. It promotes differentiation and apoptosis in acute myeloid leukemia (AML) models, particularly in combination with arsenic trioxide. This combination has been shown to induce monocytic differentiation in NB4 acute promyelocytic leukemia (APL) cells and HL-60 AML cells^4^.

**Comparable anti-proliferative effects of paricalcitol, ATRA, and 25(OH)D₃ in NB4 leukemic cell models**

The effects of ATRA, 25(OH)D₃, and paricalcitol on cell proliferation are presented in Supplementary Figure 1. Across all treatment conditions, no significant differences were observed in cell number or cell division rates. These findings suggest that paricalcitol, like ATRA and 25(OH)D₃, does not independently alter cell proliferation dynamics in NB4 leukemic cell models.


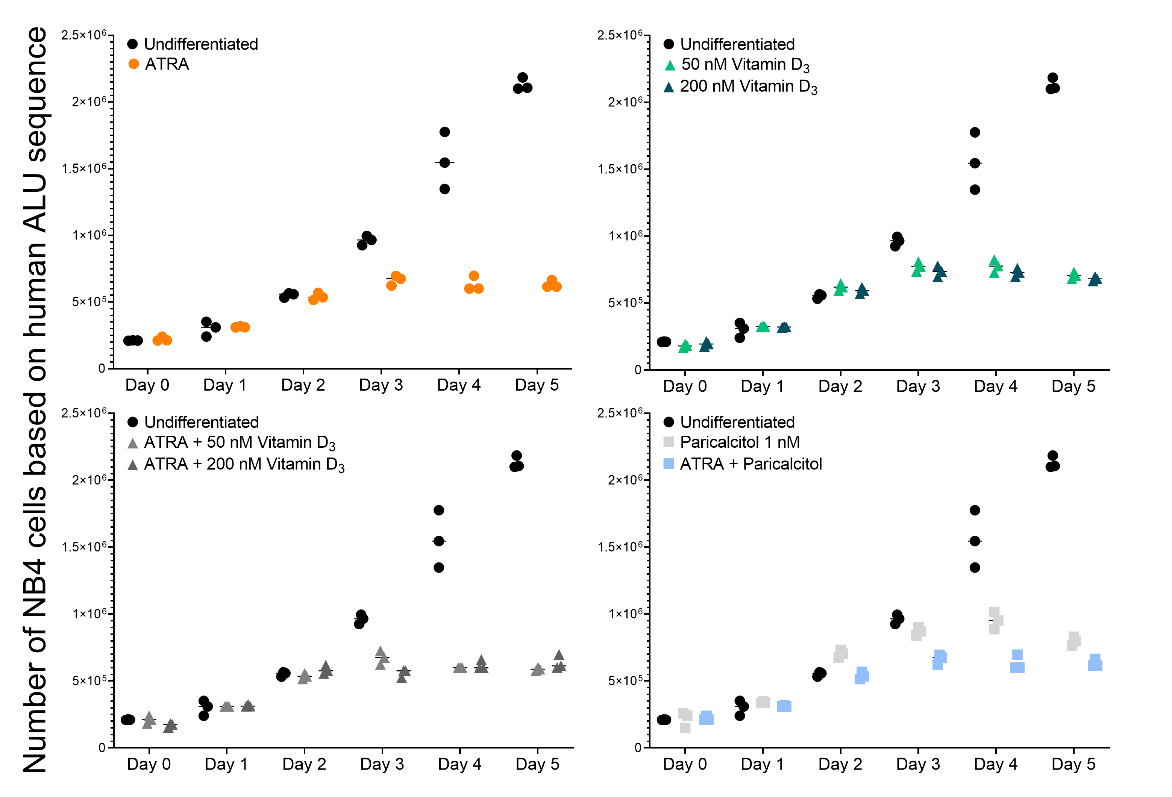


**Supplementary Figure 1. 25(OH)D3 administration reduces the activity of the NF-κB pathway without altering cell survival or cell numbers**. Cell counts were performed using human-ALU-based RT-qPCR method to count the total numbers of APL cells. The graphs show cell numbers measured in triplicates for five days (n=5), where APL cells were treated with 1 µM ATRA, 1 µM ATRA + 25(OH)D3 50 nM, 1 µM ATRA + 25(OH)D3 200 nM and 1 µM ATRA + 1 nM Paricalcitol treatment.

**ATRA plus paricalcitol treatment does not significantly reduce NF-κB reporter activity compared to ATRA alone**

Treatment of NB4 WT cells with ATRA significantly increased NF-κB luciferase reporter gene activity. Cotreatment with 25(OH)D₃ reduced this activity in a dose-dependent manner. In contrast, paricalcitol did not significantly suppress NF-κB reporter activity when combined with ATRA (Supplementary Figure 2), indicating that its inhibitory effect on NF-κB transcription may be less pronounced or mechanistically distinct in this context.


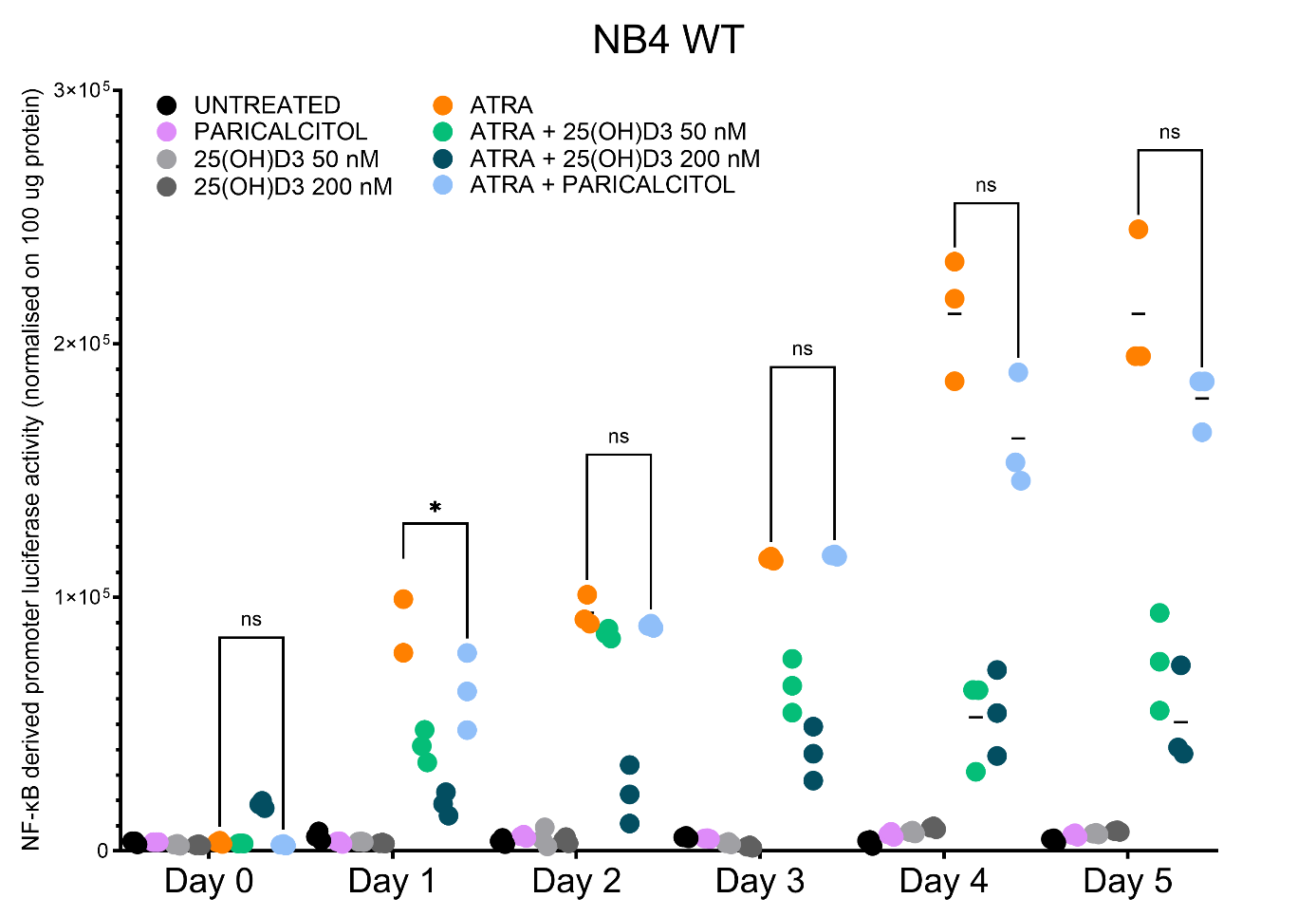


**Supplementary Figure 2. 25(OH)D3 administration reduces the activity of the NF-κB pathway without altering the cell survival or cell numbers. (A) Measurement of NF-κB response element-driven luciferase activity in NB4 WT cells treated** with 1 µM ATRA, 1 µM ATRA plus 50 and 200 nM concentrations of 25(OH)D3 or together with 1nM Paricalcitol for five days. The graph represents the mean RLU values ± S.D. (n=3). Statistical significance was determined via Two-way analysis of variance (ANOVA; Bonferroni post-hoc test, * p < 0.05, ** p< 0.01 and *** p < 0.001, **** p < 0.0001).

**The cytokine storm induced by ATRA in differentiated NB4 WT cells is silenced *in vitro* by paricalcitol, but not more effectively than by 25(OH)D₃**

The cytokine storm triggered by ATRA-induced differentiation in NB4 WT cells was significantly attenuated *in vitro* by both paricalcitol and 25(OH)D₃ (Supplementary Figure 3). However, paricalcitol did not demonstrate superior efficacy compared to 25(OH)D₃ in reducing pro-inflammatory cytokine levels, suggesting comparable anti-inflammatory potential between the two vitamin D₃ analogues under these conditions.


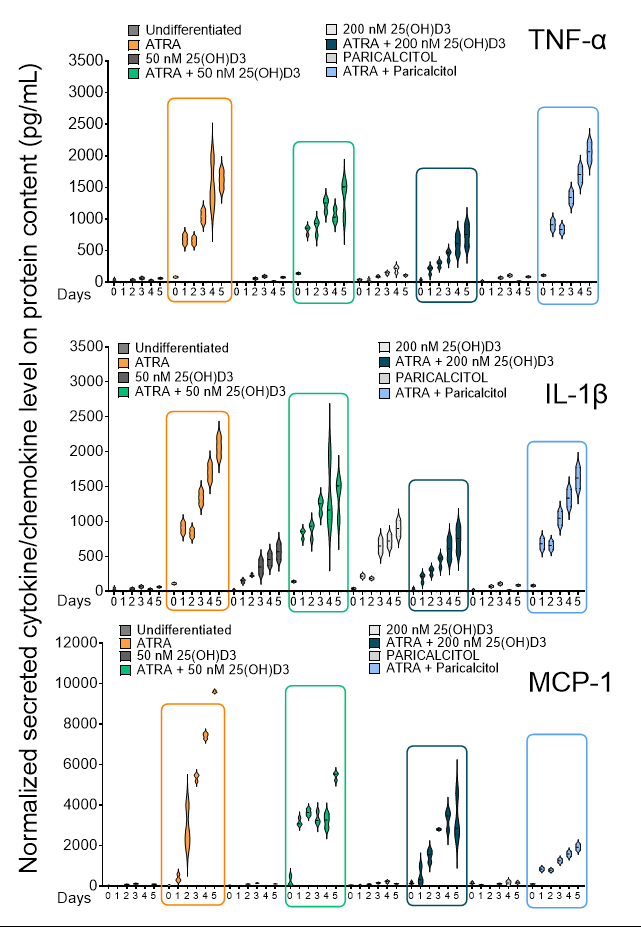


**Supplementary Figure 3. Representative ELISA data from NB4 WT cells treated under different conditions.** NB4 WT cells were treated with 1 µM ATRA, 1 µM ATRA +25(OH)D₃ 50 nM, 1 µM ATRA + 25(OH)D₃ 200 nM, and 1 µM ATRA + 1 nM Paricalcitol (a 25(OH)D₃ analogue). The colored frames indicate the different treatments over the course of the experiment.

**References**

1 Donate-Correa, J. *et al.* Selective vitamin D receptor activation as anti‐inflammatory target in chronic kidney disease. *Mediators of inflammation* **2014**, 670475 (2014).

2 Pihlstrøm, H. K. *et al.* Exploring the potential effect of paricalcitol on markers of inflammation in de novo renal transplant recipients. *Plos one* **15**, e0243759 (2020).

3 Zhang, Q., Li, M., Zhang, T. & Chen, J. Effect of vitamin D receptor activators on glomerular filtration rate: a meta-analysis and systematic review. *PloS one* **11**, e0147347 (2016).

4 Udensi, U. K. & Tchounwou, P. B. Dual effect of oxidative stress on leukemia cancer induction and treatment. *Journal of Experimental & Clinical Cancer Research* **33**, 106 (2014).
