# Supplementary material for: Anti-inflammatory 25(OH)D3, a natural steroid hormone, may complement all-trans retinoic acid therapy for differentiation syndrome in acute promyelocytic leukemia
Source: Cell Death Dis. 2025 Nov 3;16(1):787. doi: 10.1038/s41419-025-08109-7 (PMC12583587; doi:10.1038/s41419-025-08109-7)
Supplement: Supplementary file 3 — Supplementary figures [file 41419_2025_8109_MOESM3_ESM.pdf]

Supplementary Materials

Paricalcitol: a clinical vitamin D_3 analogue

Paricalcitol is a vitamin D_3 analogue used clinically, primarily in the treatment of secondary hyperparathyroidism in patients with chronic kidney disease. Substantial evidence from molecular studies, animal models, and clinical trials in chronic kidney disease, dialysis, and transplant populations indicates that paricalcitol exerts potent anti-inflammatory effects. These effects are mediated through direct activation of the vitamin D receptor (VDR), inhibition of NF-κB/p65 transcriptional activity, and suppression of key pro-inflammatory cytokines[1-3].

In addition to its anti-inflammatory properties, paricalcitol has demonstrated anti-leukemic activity. It promotes differentiation and apoptosis in acute myeloid leukemia (AML) models, particularly in combination with arsenic trioxide. This combination has been shown to induce monocytic differentiation in NB4 acute promyelocytic leukemia (APL) cells and HL-60 AML cells[4].

Comparable anti-proliferative effects of paricalcitol, ATRA, and 25(OH)D_3 in NB4 leukemic cell models

The effects of ATRA, 25(OH)D_3, and paricalcitol on cell proliferation are presented in Supplementary Figure 1. Across all treatment conditions, no significant differences were observed in cell number or cell division rates. These findings suggest that paricalcitol, like ATRA and 25(OH)D_3, does not independently alter cell proliferation dynamics in NB4 leukemic cell models.

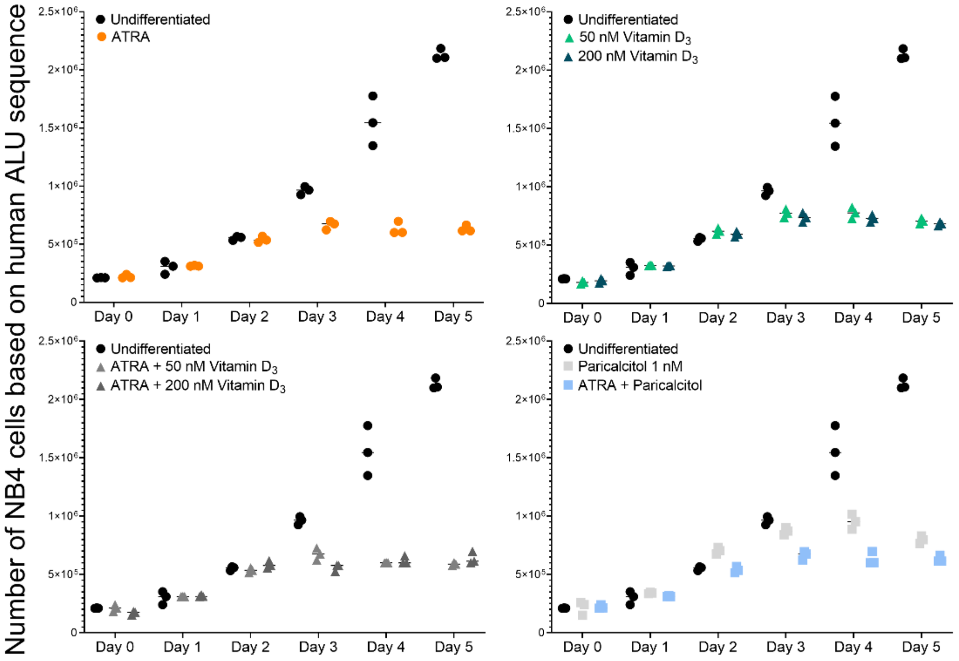

Supplementary Figure 1. 25(OH)D3 administration reduces the activity of the NF-κB pathway without altering cell survival or cell numbers. *Cell counts were performed using human-ALU-based RT-qPCR method to count the total numbers of APL cells. The graphs show cell numbers measured in triplicates for five days (n=5), where APL cells were treated with 1 µM ATRA, 1 µM ATRA + 25(OH)D3 50 nM, 1 µM ATRA + 25(OH)D3 200 nM and 1 µM ATRA + 1 nM Paricalcitol treatment.*

ATRA plus paricalcitol treatment does not significantly reduce NF-κB reporter activity compared to ATRA alone

Treatment of NB4 WT cells with ATRA significantly increased NF-κB luciferase reporter gene activity. Cotreatment with 25(OH)D_3 reduced this activity in a dose-dependent manner. In contrast, paricalcitol did not significantly suppress NF-κB reporter activity when combined with ATRA (Supplementary Figure 2), indicating that its inhibitory effect on NF-κB transcription may be less pronounced or mechanistically distinct in this context.

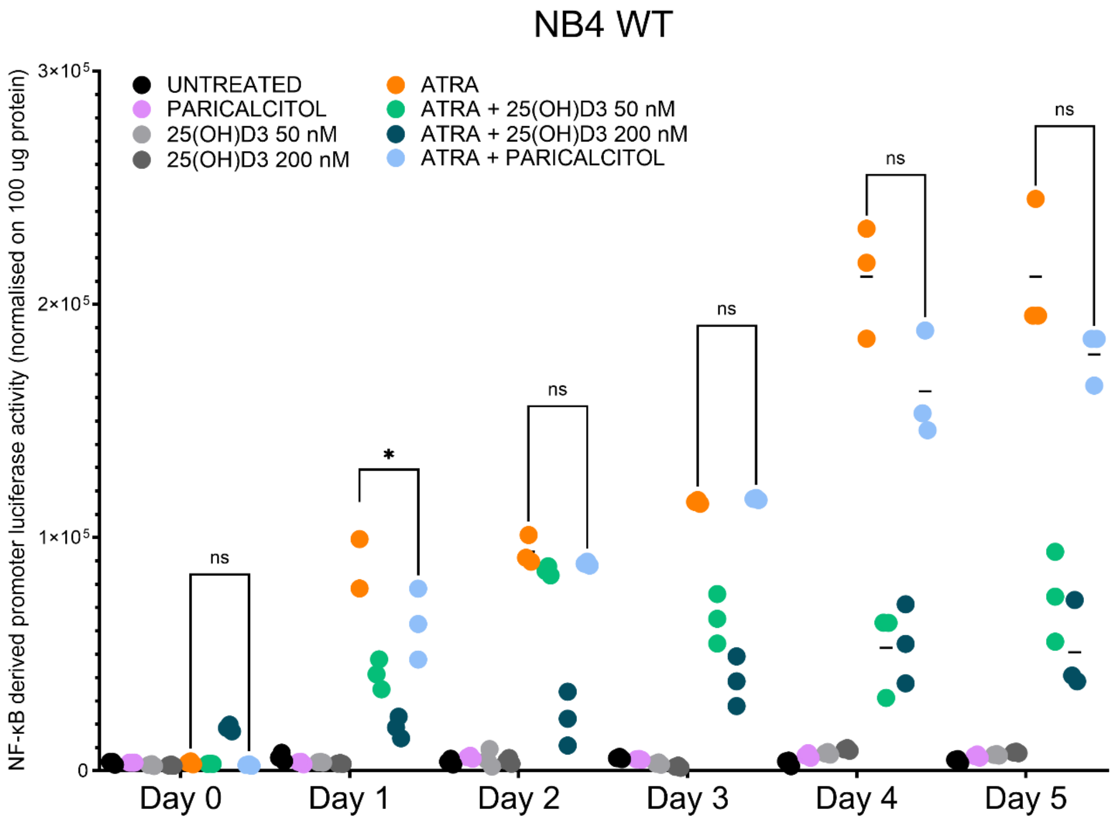

Supplementary Figure 2. 25(OH)D3 administration reduces the activity of the NF-κB pathway without altering the cell survival or cell numbers. (A) Measurement of NF-κB response element-driven luciferase activity in NB4 WT cells treated *with 1 μM ATRA, 1 μM ATRA plus 50 and 200 nM concentrations of 25(OH)D3 or together with 1nM Paricalcitol for five days. The graph represents the mean RLU values ± S.D. (n=3). Statistical significance was determined via Two-way analysis of variance (ANOVA; Bonferroni post-hoc test, * p < 0.05, ** p< 0.01 and *** p < 0.001, **** p < 0.0001).*

The cytokine storm induced by ATRA in differentiated NB4 WT cells is silenced *in vitro* by paricalcitol, but not more effectively than by 25(OH)D_3

The cytokine storm triggered by ATRA-induced differentiation in NB4 WT cells was significantly attenuated *in vitro* by both paricalcitol and 25(OH)D_3 (Supplementary Figure 3). However, paricalcitol did not demonstrate superior efficacy compared to 25(OH)D_3 in reducing pro-inflammatory cytokine levels, suggesting comparable anti-inflammatory potential between the two vitamin D_3 analogues under these conditions.

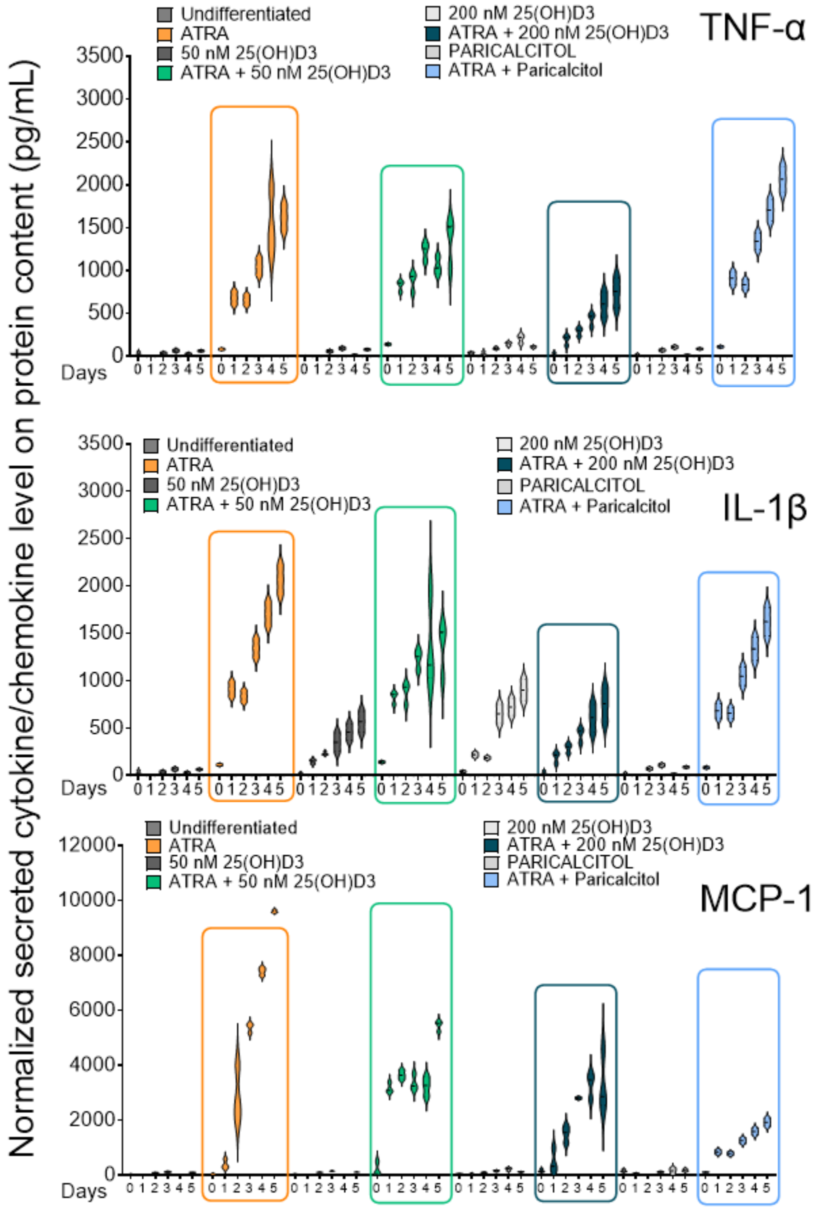

Supplementary Figure 3. Representative ELISA data from NB4 WT cells treated under different conditions. *NB4 WT cells were treated with 1 µM ATRA, 1 µM ATRA +25(OH)D_3 50 nM, 1 µM ATRA + 25(OH)D_3 200 nM, and 1 µM ATRA + 1 nM Paricalcitol (a 25(OH)D_3 analogue). The colored frames indicate the different treatments over the course of the experiment.*

References

1 Donate-Correa, J. *et al*. Selective vitamin D receptor activation as anti-inflammatory target in chronic kidney disease. *Mediators of inflammation* **2014**, 670475 (2014).

2 Pihlstrøm, H. K. *et al*. Exploring the potential effect of paricalcitol on markers of inflammation in de novo renal transplant recipients. *Plos one* **15**, e0243759 (2020).

3 Zhang, Q., Li, M., Zhang, T. & Chen, J. Effect of vitamin D receptor activators on glomerular filtration rate: a meta-analysis and systematic review. *PloS one* **11**, e0147347 (2016).

4 Udensi, U. K. & Tchounwou, P. B. Dual effect of oxidative stress on leukemia cancer induction and treatment. *Journal of Experimental & Clinical Cancer Research* **33**, 106 (2014).
